# A sweat-responsive covalent organic framework film for material-based liveness detection and sweat pore analysis

**DOI:** 10.1038/s41467-023-36291-9

**Published:** 2023-02-03

**Authors:** Qing Hao, Xiao-Rui Ren, Yichen Chen, Chao Zhao, Jingyi Xu, Dong Wang, Hong Liu

**Affiliations:** 1 State Key Laboratory of Bioelectronics, School of Biological Science and Medical Engineering, Southeast University, 2# Sipailou, Nanjing, Jiangsu 210096 China; 2 Key Laboratory of Molecular Nanostructure and Nanotechnology, Beijing National Laboratory for Molecular Sciences, CAS Research/Education Center for Excellence in Molecular Sciences, Institute of Chemistry, Chinese Academy of Sciences, Beijing, 100190 P.R. China

**Keywords:** Synthesis and processing, Polymers, Characterization and analytical techniques

## Abstract

Covalent organic frameworks have shown considerable application potential and exceptional properties in the construction of stimulus-responsive materials. Here, we designed a sweat-responsive covalent organic framework film for material-based fingerprint liveness detection. When exposed to human sweat, the COF_TPDA-TFPy_ film can transform from yellow to red. The COF_TPDA-TFPy_ film, when touched by living fingers, can produce the naked-eye-identified fingerprint pattern through the sweat-induced color change, while artificial fake fingerprints cannot. This technique, which we named material-based liveness detection, can thus intuitively discern living fingers from fake fingerprints with a 100% accuracy rate. Additionally, the distribution of sweat pores on human skin can also be collected and analyzed by shortening the contact time. By merely washing them with ethanol, all the samples can be utilized again. This work inventively accomplished material-based liveness detection and naked-eye-identified sweat pore analysis and highlighted their potential for use in clinical research and personal identification.

## Abstract (web summary)

Covalent organic frameworks are more and more used in the design of stimuli responsive materials. Here, the authors expand the application range of COFs by demonstrating a sweat induced colour change for detection of fingerprints using a sweat responsive COF.

## Introduction

Fingerprints are distinctive patterns that are personal to every individual. Fingerprints and their level 3 features, such as sweat pore distribution information^1,2^, are generally utilized as accurate standards in personal identification. Unfortunately, the existing personal identification systems based on fingerprints have often suffered attacks by artificial fake fingerprints, which pose serious hazards to individual safety and property^3,4^. Therefore, extensive effort has been put into developing fingerprint identification systems that can distinguish between living fingers and fake fingerprints, which are defined as liveness detection^3,4^. Previously, fingerprint liveness detections were generally accomplished by extracting and analyzing the patterns and level 3 features of the fingerprint images of living fingers and fake fingerprints based on sophisticated computer algorithms in the field of computer science^5–9^. From the perspective of material science, it is quite appealing to develop a type of material that could simply distinguish living fingers from fake fingerprints at the time of fingerprint collection. In this case, liveness detection would be achieved through the design of fingerprint collection materials, rather than computer software development. Realizing the liveness detection based on material science, which we defined as material-based liveness detection, may offer a practical solution to the computer science problem through a material strategy.

**Fig. 1:**
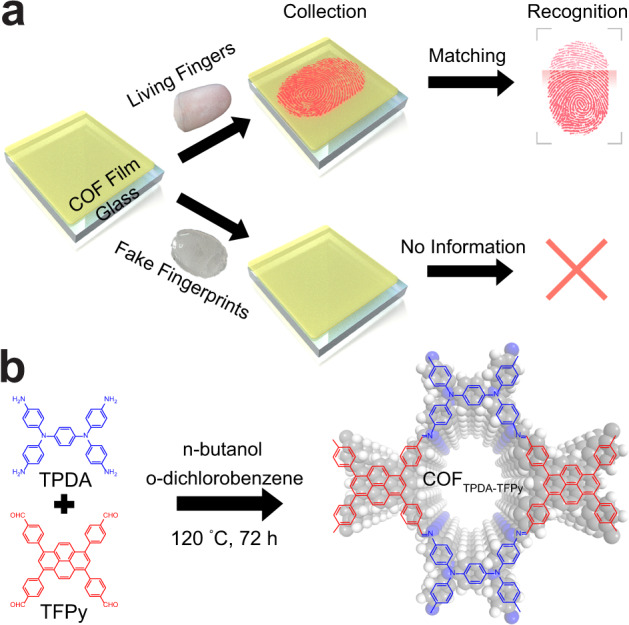
Material-based liveness detection with COF film. **a** Schematic of the material-based liveness detection based on the COF_TPDA-TFPy_ film. **b** Chemical structures of monomers and COF_TPDA-TFPy_.

One of the biggest differences between fake fingerprints and living fingers is that fake fingerprints cannot secret sweat like living fingers. Using sweat-responsive materials instead of optical sensors to collect sweat fingerprints should be possible to naturally discern between living fingers and fake fingerprints. Here, we divide the fingerprint matching process into two steps: the fingerprint collection step and the fingerprint recognition step (Fig. 1a). The previous code-based fingerprint liveness detection method occurred in the recognition step by image processing^5–9^, whereas the material-based liveness detection approach would discriminate at the fingerprint collection stage. However, there is still no research on material-based liveness detection methods till now. We believe that developing a type of sweat-responsive material, that can produce naked-eye-identified fingerprints and their level 3 feature patterns, would be a good substitute for fingerprint collection. In this situation, living fingers would generate fingerprint images on the sweat-responsive material, whereas fake fingerprints cannot supply any information, enabling the material-based liveness detection to be realized.

Covalent organic frameworks (COFs) are a class of porous organic polymers with stable chemical properties and long-range ordered crystal structures that have experienced explosive development over the past decade^10–17^. The designable chemical composition and structure confer COFs a variety of functions, and COFs have been successfully applied in the fields of gas storage and separation^18–21^, catalytic science^22–24^, chemical sensing^25–27^, energy science and electrochemistry^28–33^. Among them, stimulus-responsive chromogenic COFs, including solvatochromic^34–36^, acidochromic^37–39^, and electrochromic COFs^40–43^, have aroused growing research interest. These works highlighted the critical contribution of COFs‘ highly ordered porous crystal structures to the improvement of stimulus-response properties. For instance, the highly ordered COFs exhibit solvatochromic phenomena much better than commercial materials, with an ultra-fast response time and great reversibility^34^. We believe that the functional COF materials with a long-range ordered pore structure would show effective color change to water, organic acids, or other compositions in sweat, and achieve reusable and instrument-free fingerprint collection and analysis for material-based liveness detection.

In this work, we have achieved material-based fingerprint liveness detection based on COF film. The sweat-responsive COF_TPDA-TFPy_ film with good crystallinity was directly grown on the transparent glass substrates (Fig. 1b). When subjected to humidity streams, COF_TPDA-TFPy_ film can alter its color from yellow to red in a reversible manner, whereas sweat leaves a stable color change that does not naturally fade. After being gently touched by a human finger for around 10 s, the trace sweat secreted by sweat pores in the friction ridge caused a steady color change in the contact area of the COF_TPDA-TFPy_ film. Then, a naked-eye-identified sweat-induced fingerprint was left on the COF_TPDA-TFPy_ film, while the fake fingerprints left no imprint (Fig. 1a). Sweat-induced fingerprints from the same donor can be easily matched in the database by fingerprint matching software, and 100% of the attacks of fake fingerprints can be blocked before the recognition step. Therefore, material-based liveness detection was naturally realized with a 100% accuracy rate in the sight of material science. Additionally, by reducing the contact time (for around 1~5 s), the sweat pore distribution information from fingerprints and other parts of the body can also be gathered. Since many diseases (such as dermatosis and endocrinosis) may be associated with sweat secretion behavior, this sensitive analysis method may provide information on the distribution and sweat secretion behavior of sweat pores, and thus may provide a perspective for clinical research in the future. All of these samples can be quickly and easily collected, evaluated, and reused more than 50 times by washing with ethanol without the need for large instruments. We believe that this work would provide a unique sight to the field of personal identification based on material science and would have a broad range of potential applications.

## Results and discussion

### Preparation and characterization of COF_TPDA-TFPy_ powders and films

Typically, the COF_TPDA-TFPy_ powders were synthesized through the solvothermal method at 120 °C for 72 h, taking the o-dichlorobenzene (o-DCB) and n-butanol (n-BuOH) mixture as solvent. N, N, N’, N’-tetrakis(4-aminophenyl)−1,4-benzenediamine (TPDA) and 1,3,6,8-Tetrakis(4-formylphenyl)pyrene (TFPy) were chosen as building blocks, and 6 M acetic acid (HOAc) aqueous as catalyst (Fig. 1b). A number of strong reflections were observed in the powder X-ray diffraction (PXRD) pattern of COF_TPDA-TFPy_ powders at 5.41° (110), 7.59° (200), 10.81° (220), 12.0° (310), and 20.9° (001). Following Pawley refinement, the P2/m symmetric structural model of COF_TPDA-TFPy_ offers well fit for these experimental results. The unit cell parameters were calculated as a = 2.35 nm, b = 2.28 nm, c = 0.43 nm, α = β = 90°, γ = 78°, respectively (Supplementary Figs. 1, 2; Table 3). Meanwhile, Fourier-transform infrared spectroscopy (FTIR) and ^13^C nuclear magnetic resonance (NMR) spectra of COF_TPDA-TFPy_ were also measured. The peak in the FTIR spectra of COF_TPDA-TFPy_ at around 1630 cm^−1^ revealed the stretching mode of the imine bonds (-C = N). At the same time, the decreasing of peaks at around 1690 cm^−1^ (characteristic peak of the -CHO groups) and around 3200 cm^−1^ (characteristic peaks of the Ph-NH_2_ groups) further confirmed the occurrence of the Schiff bases reactions (Supplementary Fig. 3). Additionally, the chemical shift at roughly 153 ppm (carbon g) in the ^13^C NMR spectra strongly corroborated the successful synthesis of imine bonds, while all the other carbon resonances can be assigned to the chemical structure of COF_TPDA-TFPy_ (Supplementary Fig. 4). The Brunauer–Emmett–Teller (BET) experiment data showed a nitrogen adsorption Type-I isotherm with a sharp step at P/P_0_ < 0.01, which supports the micropore structure of COF_TPDA-TFPy_ (Supplementary Fig. 5a)^44^. The pore size distribution (PSD) profiles obtained using non-local density functional theory revealed a very narrow pore size distribution with a maximum at 1.32 nm, which fit well with the atomistic model of COF_TPDA-TFPy_ (Supplementary Fig. 5b). These results demonstrated that CO_FTPDA-TFPy_ powders were successfully prepared.

**Fig. 2:**
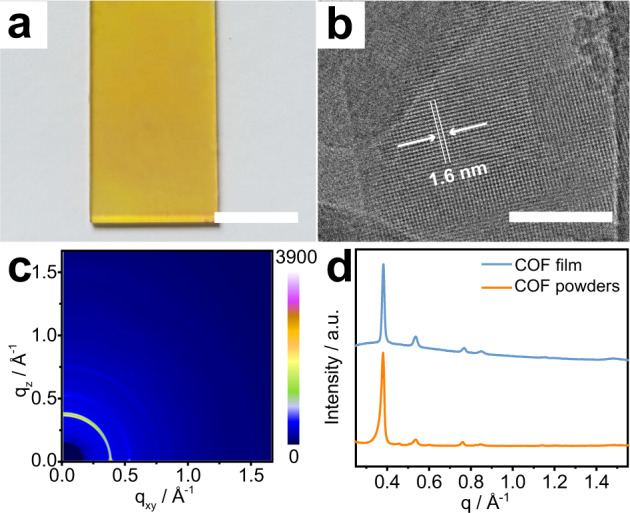
Characterization of COF_TPDA-TFPy_ film. **a** Photograph of COF_TPDA-TFPy_ film. (Scale bar: 1 cm) **b** TEM images of COF_TPDA-TFPy_ film. (Scale bar: 50 nm) **c** GIWAXS data of the COF_TPDA-TFPy_ film on glass substrates. **d** Projections of GIWAXS data of COF_TPDA-TFPy_ films near q_z_ = 0 (Blue) and PXRD data of COF_TPDA-TFPy_ powder (Orange).

To facilitate subsequent study, the COF_TPDA-TFPy_ films were also directly grown on the transparent glass using the solvothermal method (See “Methods” section). The as-prepared COF_TPDA-TFPy_ films displayed a consistent yellow morphology (Fig. 2a). The images of scanning electron microscopy (SEM) and atomic force microscopy (AFM) revealed that the COF_TPDA-TFPy_ film was formed by the stacking of small quadrilateral nanosheets with a thickness of around 100 nm (Supplementary Figs. 6, 7). The transmission electron microscopy (TEM) images were collected by scraping the film from the substrate. As shown in Fig. 2b, the vast area of periodic pore pattern was observed on the COF_TPDA-TFPy_ nanosheets. The pore structure is consistent well with the predicted structure of COF_TPDA-TFPy_ and showed a pore-to-pore period of around 1.6 nm. (Supplementary Figs. 8, 9). Meanwhile, the 2D synchrotron radiation grazing incidence wide-angle X-ray scattering (GIWAXS) data of COF_TPDA-TFPy_ film was also recorded. The Bragg peaks appeared as diffraction rings (Fig. 2c), and the projections of the data sets near q_z_ = 0 gave the diffraction peaks at 0.38 (110), 0.54 (200), 0.77 (220), 0.85 (310), and 1.48 Å^−1^ (001), which could agree well with the PXRD of COF_TPDA-TFPy_ powders (Fig. 2d). These results verified the successful synthesis of the COF_TPDA-TFPy_ film.

### Material-based liveness detection of fingerprints

**Fig. 3:**
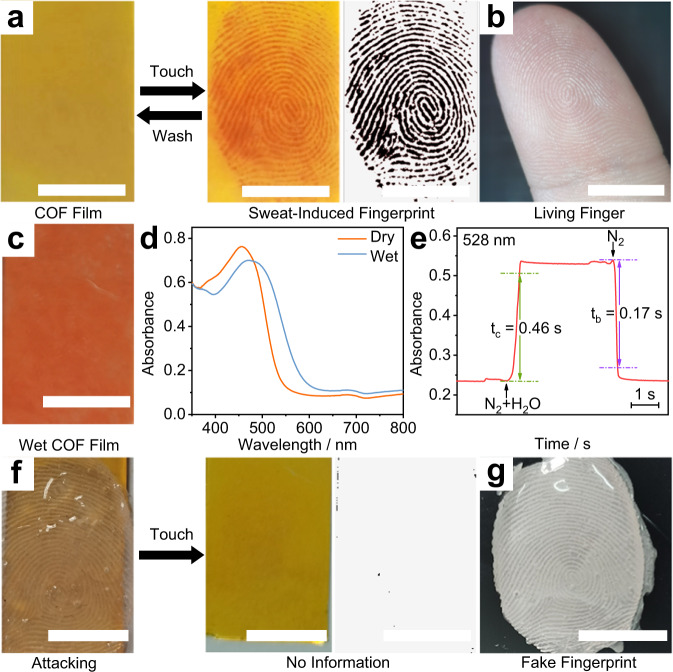
Sweat-induced fingerprint collected by COF_TPDA-TFPy_ film. **a** Photograph of COF_TPDA-TFPy_ film before and after the touch of human fingers for 10 s taken by a smartphone. The image with grey background is the sweat-induced fingerprint extracted using Photoshop. The black colored images are added pseudocolors by a Photoshop program for clear display. **b** Photographs of a living finger. **c** Photographs of COF_TPDA-TFPy_ film at wet (N_2_ + H_2_O) condition. **d** Corresponding absorption spectra at dry and wet conditions, respectively. **e** Response time of COF_TPDA-TFPy_ film at 528 nm by alternately exposing the COF_TPDA-TFPy_ film to wet and dry N_2_ stream. **f** Photographs of COF_TPDA-TFPy_ film attacked by artificial fake fingerprint. **g** Corresponding artificial fake fingerprint from the finger in (**b**). (Scale bars: 1 cm).

Touching the COF_TPDA-TFPy_ film with a living finger for 10 s would result in a naked-eye-identified dark-red fingerprint impression on the yellow film (Fig. 3a, b; Supplementary Movie 1). At first, we believed this phenomenon can be attributed to the hydrochromism of COF. Actually, the COF_TPDA-TFPy_ films do exhibit remarkable hydrochromic phenomena. As shown in Fig. 3c, exposing the COF_TPDA-TFPy_ film to humidity N_2_ stream results in a color change from yellow to red. The humidity breathing also showed a similar phenomenon (Supplementary Movie 2). However, the color change of COF_TPDA-TFPy_ film caused by water is totally reversible after drying, while the sweat-resulted pattern would not disappear naturally. Therefore, although the color change is similar, we believe that the sweat-induced color change comes from both water and other complex components of sweat, rather than simple hydrochromism.

We then experimented with several typical sweat compositions. With humidity stream stimulation for the water initially, the absorbance peak of COF_TPDA-TFPy_ film, which was previously at about 457 nm in the absorption spectra, migrated to about 480 nm (Fig. 3d). The previous work^34^ suggested that solvent-induced electronic structure change is responsible for the solvatochromic characteristics of COFs. The hydrochromic features brought on by electronic processes would be encouraged by the periodic donor-acceptor pairings in the COF skeleton. Consequently, the COF_TPDA-TFPy_ film containing intramolecular donor-acceptor pairs resulted in a pronounced electronic structure-based hydrochromic phenomenon (Supplementary Fig. 10). The absorbance value at 528 nm, the wavelength displayed the highest variation (Supplementary Fig. 11), switched quickly as the N_2_ stream alternated between the dry and humid states (Fig. 3e). The ultra-fast coloring time (t_c_) and bleaching time (t_b_) at 528 nm, where the absorbance value changed by more than 90%, were estimated as 0.46 s and 0.17 s, respectively. Meanwhile, within 50 switching cycles, the absorbance value kept switching steadily (Supplementary Fig. 12). Even if there are minor changes in the absorption spectra after 200 cycles, it can essentially return to its previous state after being washed with ethanol (Supplementary Fig. 13).

For other components, the COF_TPDA-TFPy_ film was treated by solutions with some common ingredients in sweat. Some acids can produce a grey tint, which may be caused by COF protonation. (Supplementary Table 1). And some compositions like some protein (e.g. dermcidin, one of the most abundant proteins in sweat^45^) solutions could generate a stable red area (Supplementary Fig. 14; Table 1). We supposed that the moisture in proteins from the sweat and the water-holding capacity of these proteins may provide a stable humidity micro-environment in the fingerprint residue^46,47^ left on the COF_TPDA-TFPy_ film. After contact, the FTIR spectrum of the COF_TPDA-TFPy_ pellet showed rising -OH bands, which also supported the presence of water residue (Supplementary Fig. 15). Considering that the color and spectral changes caused by sweat are similar to those caused by water, we speculate that the synergistic interaction of these components in sweat resulted in the long-term sweat-responsive deep red color.

**Table 1:** Similarity factors of 12 fingerprint samples from one donor with the fingerprint sample in Fig. 4a

Sample	Similarity factor	Sample	Similarity factor	Sample	Similarity factor
101_1	0.78791	101_5	0.73	102_1	1
101_2	0.85896	101_6	0.74726	102_2	0.81111
101_3	0.81698	101_7	0.69437	102_3	0.84327
101_4	0.86518	101_8	0.80556	102_4	0.76422

Washing with ethanol is a straightforward method for erasing these stable images (Fig. 3a). Despite some unavoidable wear and tear, after 50 cycles of touch-and-wash operations, the COF_TPDA-TFPy_ film can still provide clear enough images (Supplementary Fig. 16). The good reusable ability makes the per-collection cost less than 0.02 dollars.

Note that such sweat-induced fingerprints can only be derived from the sweat secretion of living fingers, the artificial fake fingerprint (Supplementary Fig. 17) that cannot secret sweat did not generate any imprints (Fig. 3f, g). However, the artificial fingerprint can mimic a living finger to generate a realistic-looking fingerprint with colored inks (Supplementary Fig. 18), which can pose a great threat to personal safety and personal property safety. Therefore, the COF_TPDA-TFPy_ film can simply and directly collect sweat-induced fingerprints, and at the same time distinguish between living fingers and fake fingerprints. As we proposed above, the COF-based fingerprint collection and recognition method can be defined as material-based liveness detection. In this scenario, fake fingerprint attacks are unable to produce any meaningful patterns and can therefore be blocked as early as the collection step rather than the following recognition step.

**Fig. 4:**
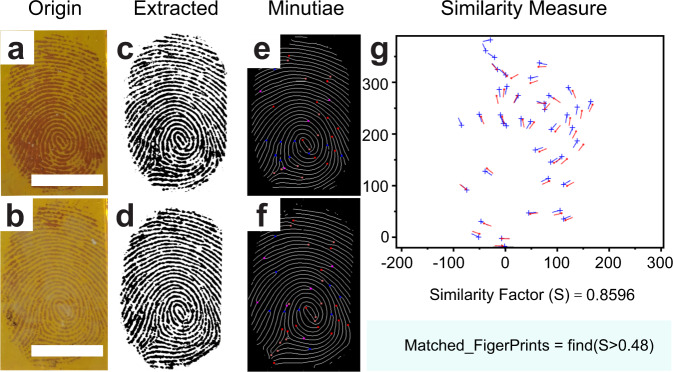
Sweat-induced fingerprint recognition step. **a**, **b** Photographs of two sweat-induced fingerprints. **c**, **d** Images extracted from (**a**, **b**), respectively. The black (**c**, **d**) colored images are added pseudocolors by a Photoshop program for clear display. **e**, **f** Minutiae images calculated from (**c**, **d**). **g** The result of the similarity measure with a similarity factor of 0.8596 (Scale bars: 1 cm).

The fingerprint patterns collected by COF_TPDA-TFPy_ film were highly compatible with the existing fingerprint recognition software. We use a simple approach fingerprint matching code created by V. K. Alilou^48^ to test fingerprint recognition and the accuracy of material-based liveness detection. As shown in Fig. 4, the minute details in the two fingerprint samples from the same donor are identical, and the similarity factor was calculated to be 0.8596 (>0.48 would be used to confirm that the samples are from the same person). At the same time, the fake fingerprint left no information on the film and the software was inoperable.

Another 12 fingerprint samples (Number 101_1 ~ 102_4) collected by COF_TPDA-TFPy_ film from the same donor (Supplementary Data 1) have also been added to the database with a total fingerprint sample of 72 (From FVC2002^49^, number 102_5 ~ 109_8). The 12 fingerprints belonging to the same donor can be fully identified through database comparison (Table 1), and the 60 other fingerprint samples can also be fully discriminated (Supplementary Table 2). At the same time, none of the fake fingerprint attacks had any meaningful outcomes. This indicated that the material-based liveness detection method can completely block the fake fingerprint at the collection step time, which provides a totally different solution notion from that of computer science^3–9^. In contrast to the computer science method, in which the liveness detection steps are carried out after the image collection step, this material-based liveness detection occurs as early as the collection step rather than the subsequent recognition step. Be aware that fake fingerprints cannot produce useful information and do not require the subsequent fingerprint-matching procedure. Here, material-based liveness detection can be accomplished in a straightforward manner and from the perspective of material science.

### Sweat pore analysis

**Fig. 5:**
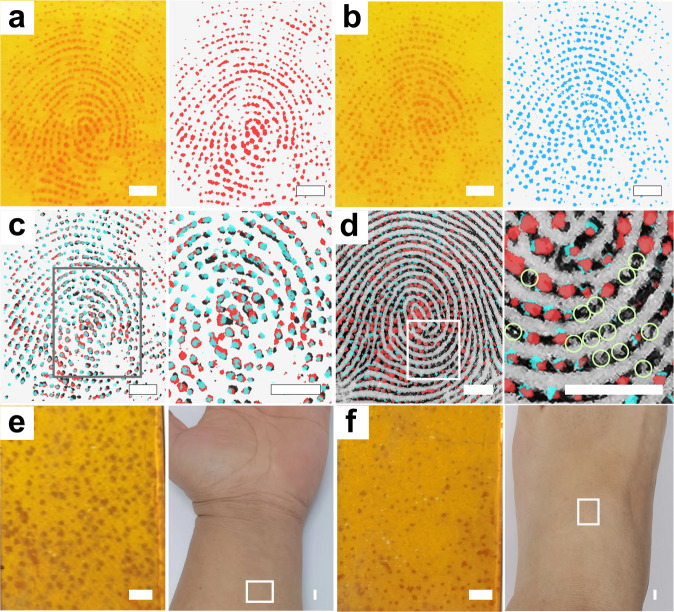
Sweat pore images collected by COF_TPDA-TFPy_ films. **a**, **b** Picture of 2 independent sweat pore distribution images and extracted sweat pore distribution images on 2 individual COF_TPDA-TFPy_ films from the same donor (Taken with a smartphone) **c** Superimposed images of 3 sweat pore images. **d** Superimposed image of sweat pore distribution images on a graphite fingerprint image. The green circles showed the non-active sweat pores. **e** Sweat pore distribution images collected from the inside of the arm. **f** Sweat pore distribution images collected from the instep (Scale bars: 20 mm). The red (**a**, **c**, **d**), blue (**b**, **c**, **d**), and black (**c**) colored images are added pseudocolors by a Photoshop program for comparison purposes.

In addition, reducing the contact time to around 1 s with human fingers can generate a red color dot pattern that matches the sweat pores on the finger (Fig. 5a, b; Supplementary Movie 3). The sweat pore information is one of the level 3 features of fingerprints, which would be valuable for personal identification based on normal or partial fingerprints^1,2^. Moreover, the detection of active sweat pores is another liveness detection method in computer science^3–9^. The sweat pores images on COF_TPDA-TFPy_ film can be directly identified by the naked eye, and easily collected just by taking photos with smartphones and using Photoshop to extract and analyze the pattern conveniently. This is different from the previous sweat pore collection methods based on solvatochromic fluorescence^50–53^, mass spectrometry^54^, electrochemiluminescences^55^, and scanning electrochemical microscopy^56^ that need the help of additional instruments. For ordinary persons without prior instrument operation experience, the instrument-free method makes data gathering and processing convenient. Moreover, the sweat pore distribution images generated by COF_TPDA-TFPy_ film can also be considered as a material-based liveness detection feature to avoid attacks of fake fingerprints in the future.

Three different fingerprints were taken on three COF_TPDA-TFPy_ films in order to assess the reproducibility of the COF-based sweat pore distribution imaging approach (Fig. 5a, b; Supplementary Fig. 19). The sweat pore images of the three samples were extracted and superimposed (Added pseudocolors). As shown in Fig. 5c, the three samples are in good agreement. The precise identification and location of non-active sweat pores can also be accomplished by superimposing the active sweat pore distribution images on the fingerprint obtained using graphite. As shown in Fig. 5d, the green circles display the non-active sweat pores that did not secret sweat.

In addition to the hairless finger, we also attempted to collect data on the distribution of sweat pores from other body areas, such as the instep, the inside of the arm with imperceptible villi, and the hairy outside of the arm (Fig. 5e, f; Supplementary Fig. 20). On the instep and the inner side of the arm, the irregularly dispersed sweat pore patterns can be directly collected (Fig. 5e, f). When collecting the hairy outside of the arm, the hair sometimes affects image acquisition (Supplementary Fig. 20b). But following shaving, similar sweat pore distribution patterns can be gathered on the hairy arm’s outer skin surface. (Supplementary Fig. 20c). In contrast to the fingers, which have friction ridges, other body parts have an irregular distribution of sweat pores. Therefore, even after prolonged contact (>10 s), the sweat pores photos will still show a scattered dot pattern rather than lines like fingerprints.

**Fig. 6:**
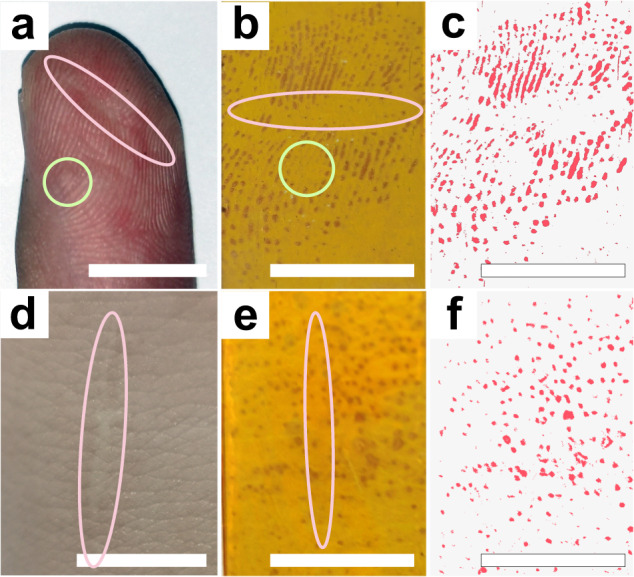
Sweat pore images of scars and keratosis. **a** Photograph of a finger with scars (red circle) and mild keratosis (green circle). **b** Sweat pore images collected from the finger in (**a**). **c** Extracted sweat pore images from (**b**). **d** Photograph of an area of the inside of an arm with scars (red circle) **e** Sweat pore images collected from the area in (**d**). **f** Extracted sweat pore images from (**e**). (Scale bars are 1 cm) The red (**c**, **f**) colored images are added pseudocolors by a Photoshop program for clear display.

This simple and rapid strategy for visualization and localization of sweat pores may have potential value in physiological and pathological studies. For example, a contributor had both scars and mild keratosis on his fingers (Fig. 6a). The image collected by COF films revealed that the center of the healed scar still lacks the ability for sweat pores to operate normally. Additionally, even while active sweat pores can be seen on keratinized skin, these pores have much lower sweat secretion capacities than the sweat pores around them, which may be a marker of local lesions or one of their causes (Fig. 6b, c). We also discovered that the sweat pores in the center of the scar on the arm did not regenerate even after healing, similar to what was seen on the fingers (Fig. 6d–f). Given that many diseases result in abnormal sweating, information about the distribution and ratio of active versus inactive sweat pores may act as useful supporting data for clinical research in the future.

In summary, we have demonstrated fingerprint material-based liveness detection based on a sweat-responsive COF film. The COF_TPDA-TFPy_ film exhibited good crystallinity and reversible hydrochromic phenomena between yellow and red, and sweat can produce a long-term color change. Living fingers touching the COF_TPDA-TFPy_ film for 10 s can generate naked-eye-identified sweat-induced fingerprints, whereas the artificial fake fingerprints cannot provide meaningful images. Therefore, while collecting fingerprint images, the COF_TPDA-TFPy_ film can naturally distinguish living fingers from fake fingerprints. Simply taking photos with smartphones can capture all of the fingerprint images, which can then be examined by fingerprint-matching software. This method is capable of 100% fingerprint recognition and 100% fake fingerprint identification in the way of material science, and we defined it as material-based liveness detection. By washing with ethanol, repeated collections can be made more than 50 times. Additionally, the sweat pore distribution images of human skin can also be collected. This method may also be used to study sweating behavior and pathological alterations to the skin’s surface. In the future, we will continue to encourage the use of this COF film-based analytic technique in the fields of clinical medical research and safe personal identification.

## Methods

### Materials and chemicals

The monomers, N, N, N’, N’-tetrakis(4-aminophenyl)−1,4-benzenediamine (TPDA, 95%) and 1,3,6,8-Tetrakis(4-formylphenyl)pyrene (TFPy, 95%) were supplied by Chemsoon Co., Ltd. Acetic acid (HOAc, 99.5%) was bought from TCI Co., Ltd. Solvents including 1,2-dichlorobenzene (o-DCB, 99 + %), 1-Butanol (n-BuOH, 99.5%) and dimethylformamide (DMF, 99.5%) were bought from Acros Organics Co., Ltd. Normal solvents like tetrahydrofuran (THF, AR) and ethanol (AR) were bought from Nanjing Chemical Reagent Co., Ltd. (Nanjing), and Aladin Co., Ltd. Ultrapure water (≥18 MΩ, Milli-Q, Millipore) was used throughout the experiment. All chemicals were used without further purification.

### Preparation of COF_TPDA-TFPy_ powders

Typically, the TPDA (47.3 mg, 0.10 mmol) and TFPy (61.8 mg, 0.10 mmol) were charged in a high vacuum thick wall reaction bottle (15 mL of volume, ϕ_in_ = 2.0 cm), the 1,2-dichlorobenzene (3 mL) and 1-butanol (3 mL) were added as mixture solvent. Then, 0.4 mL of 6 M HOAc was added to the solution and the mixture was sonicated for 30 minutes. After a traditional degassing process with three freeze-pump-thaw cycles, the reaction bottle was sealed off and left until room temperature. The sealed reaction bottle was subsequently heated and kept at 120 °C for 72 h. The resulting precipitates were collected by filtration and washed with water, DMF, and THF 3 times each. Then, COF_TPDA-TFPy_ powders were further activated by Soxhlet extraction with tetrahydrofuran for 12 h. Finally, the COF_TPDA-TFPy_ powders were dried under vacuum at 70 °C for 24 h.

### Preparation of COF_TPDA-TFPy_ film

For the preparation of COF_TPDA-TFPy_ film, the process is the same as that for the synthesis of COF_TPDA-TFPy_ powders, except that the TPDA and TFPy were reduced to 14.2 mg (0.03 mmol) and 18.6 mg (0.03 mmol), respectively. Transparent glass substrates were cut into 1.5 cm × 4.5 cm and immersed into the mixture in the reaction bottle vertically. The resulting COF_TPDA-TFPy_ films were rinsed with DMF and THF 3 times each and immersed in 75% ethanol before investigation.

### Absorption spectra measurements

In-situ absorption spectra were monitored by an Agilent Technologies Cary 60 UV-Vis spectrophotometer. For the hydrochromic investigation operation, a COF_TPDA-TFPy_ film on the glass substrate was directly put in a quartz cuvette, and a gas tube (ϕ_in_ = 0.8 cm) of a home-build gas flow control system was put on the COF_TPDA-TFPy_ film. In the two gas paths in the control system, the dry N_2_ can directly pass or through the gas washing bottle with water, respectively. The switching of the dry/wet N_2_ stream is realized by switching the gas path valve in the system.

### Fingerprints and sweat pores collection

The COF_TPDA-TFPy_ films are taken from the 75% ethanol and put in a box with silica-gel driers to make sure they exhibited a yellow appearance. The human fingers were first cleaned with 75% ethanol wipes and then fully dried with a tissue. Fingerprints were collected by softly touching on the COF_TPDA-TFPy_ film using living fingers for ~10 s and sweat pore images were collected from fingers or other parts of the body for 1~5 s respectively. The as-collected samples can be identified directly by the naked eye and obtained by taking pictures using a smartphone (Honor V30). The exact fingerprint images and distribution information of sweat pores can be extracted by Photoshop and added pseudo colors.

### Inclusion & ethics

All experiments were carried out in strict compliance with the relevant laws and with the approval of the Scientific Ethical Committee of the School of Biological Sciences and Medical Engineering, Southeast University. All the human research participants have signed a consent form.

### General characterization

PXRD patterns were collected by a PANalytical Empyrean Diffractometer with Cu Kα radiation (*λ* = 1.5416 Å) ranging from 3.5° to 35° with a speed of 1°/min at ambient temperature operated at 40 kV and 40 mA. The morphology of COF_TPDA-TFPy_ film was studied by Scanning Electron Microscope (Zeiss Ultra Plus Field Emission Scanning Electron Microscope) and Atomic Force Microscope (Bruker Multimode 8). Themis 300 was employed to obtain the Transmission Electron Microscopy images using low-dose techniques at an accelerating voltage of 300 kV. Fourier transform infrared spectroscopy (FTIR) spectra were collected by a Thermo Scientific Nicolet 5700 instrument. ^13^C cross-polarization magic angle spinning nuclear magnetic resonance (^13^C CP/MAS NMR) spectra were collected by a Bruker AVANCE III 400 NMR spectrometer. 2D Grazing incidence wide-angle X-ray scattering (GIWAXS) data were collected at 1W1A endstation, Beijing Synchrotron Radiation Facility (*λ* = 1.5496 Å), using a MarCCD (mar345) with around 438 mm from the samples to CCD.

## Supplementary information

Supplementary Information

Description of Additional Supplementary Files

Supplementary Data 1

Supplementary Movie 1

Supplementary Movie 2

Supplementary Movie 3

## Data Availability

The data that support the findings of this study are available in the paper and its supplementary information files, or available from the corresponding author upon request. The fingerprint data that support the findings of this study are available from FVC2002, http://bias.csr.unibo.it/fvc2002.

## Code availability

The code (FingerPrint Matching: A simple approach) for fingerprint analysis associated with the current submission is available at https://github.com/alilou63/fingerprint, (Created by Vahid K. Alilou).
