# Supplementary material for: A sweat-responsive covalent organic framework film for material-based liveness detection and sweat pore analysis
Source: Nat Commun. 2023 Feb 3;14:578. doi: 10.1038/s41467-023-36291-9 (PMC9894872; doi:10.1038/s41467-023-36291-9)
Supplement: Supplementary file 1 — Supplementary Information [file 41467_2023_36291_MOESM1_ESM.pdf]

Supplementary Information

A sweat-responsive covalent organic framework film for material-based liveness detection and sweat pore analysis

Qing Hao,[1]* Xiao-Rui Ren,[2] Yichen Chen,[1] Chao Zhao,[1] Jingyi Xu,[1] Dong Wang,[2]* Hong Liu[1]*

[1]State Key Laboratory of Bioelectronics, School of Biological Science and Medical Engineering,
Southeast University, 2# Sipailou, Nanjing, Jiangsu, 210096, China

[2]Key Laboratory of Molecular Nanostructure and Nanotechnology, Beijing National Laboratory for Molecular Sciences, CAS Research/Education Center for Excellence in Molecular Sciences,
Institute of Chemistry, Chinese Academy of Sciences, Beijing 100190, P.R. China
*Corresponding author. E-mail: haoq@seu.edu.cn; wangd@iccas.ac.cn; liuh@seu.edu.cn

Supplementary Figures

Characterization of $COF_{TPDA\text{-}TFPy}$

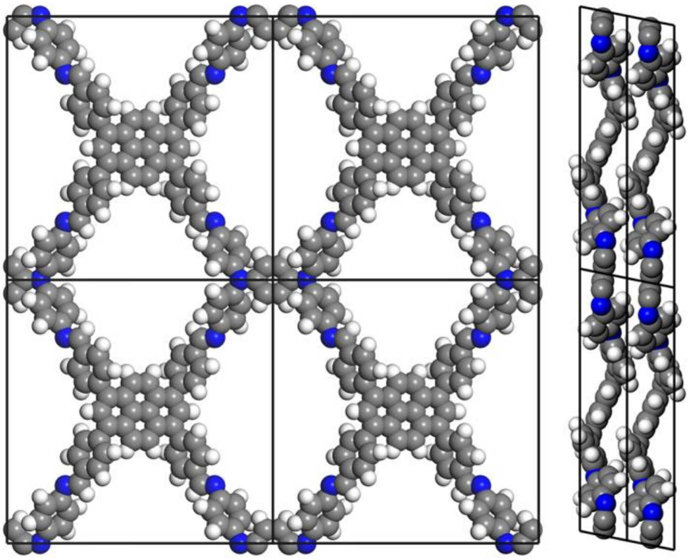

Supplementary Fig. 1. Simulated structure of the $COF_{TPDA\text{-}TFPy}$. The simulation of $COF_{TPDA\text{-}TFPy}$ was carried out using Cambridge Sequential Total Energy Package (CASTEP).

The pore-to-pore repeat distance of this model is around 1.6 nm. The predicted pore sizes of this model are 1.8 nm and 1.6 nm (corner-to-corner), 1.3 nm and 1.2 nm (bridge-to-bridge), respectively.

Powder X-ray diffraction (PXRD)

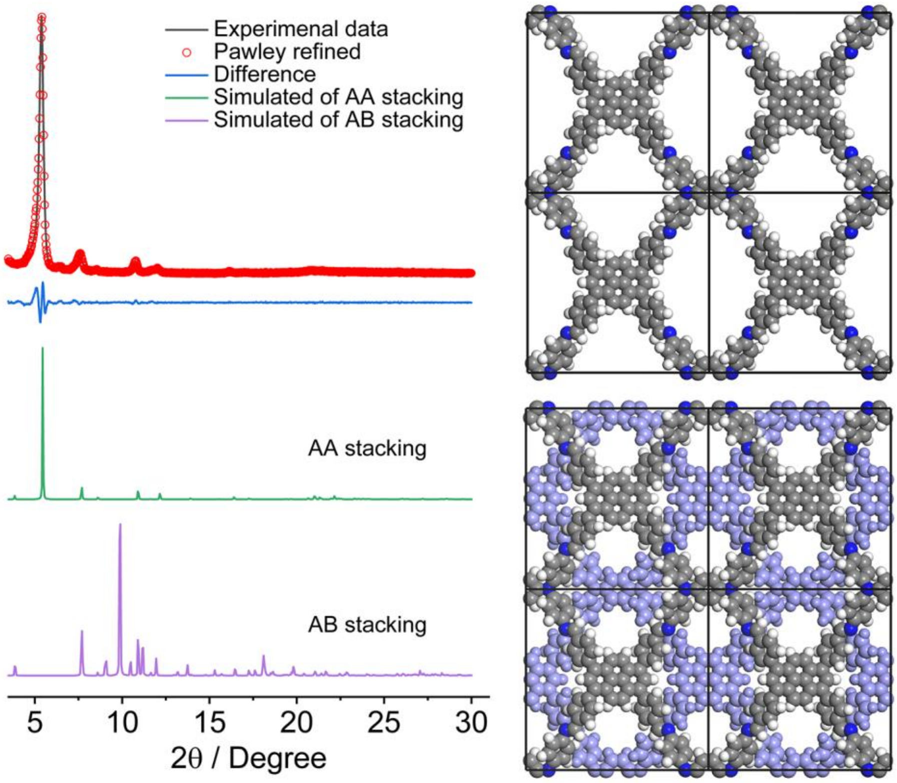

Supplementary Fig. 2. PXRD of COF$_{\text{TPDA-TFPy}}$. Powder X-ray diffraction of COF$_{\text{TPDA-TFPy}}$: the experimental data (black), the Pawley refined profile (red dot), the difference plot (blue), the calculated PXRD pattern of the AA stacking model (green), and AB stacking model (purple).

Fourier transform infrared spectroscopy (FTIR) spectra

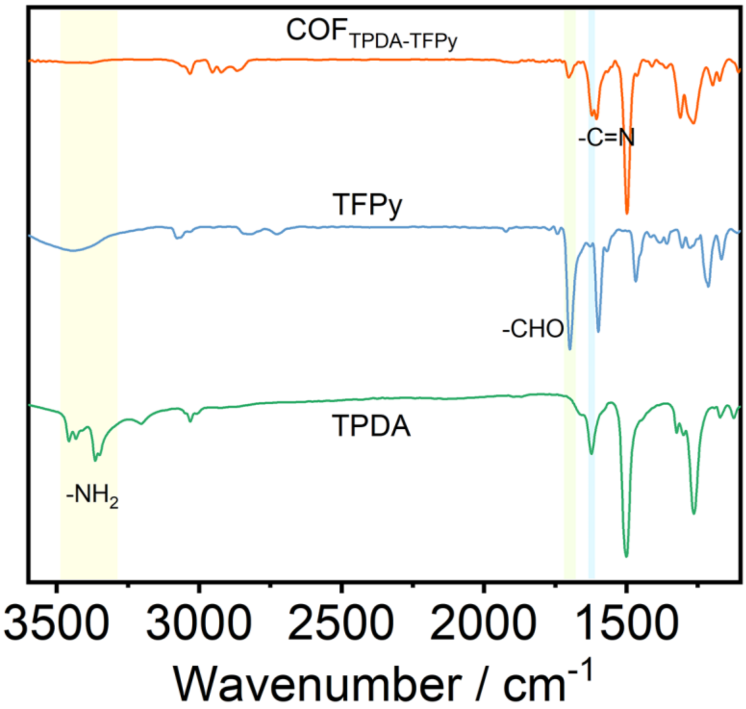

Supplementary Fig. 3. FTIR of COF$_{TPDA-TFPy}$. Comparison of FTIR spectra of COF$_{TPDA-TFPy}$ powders, TFPy, and TPDA.

^{13}C Nuclear Magnetic Resonance (NMR) spectra

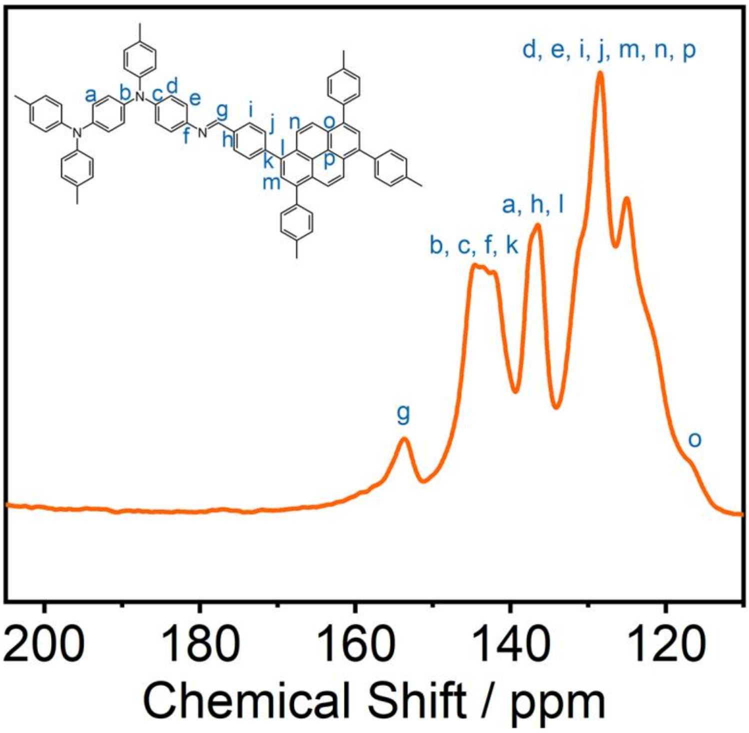

Supplementary Fig. 4. **^{13}C NMR spectra of $COF_{TPDA-TFPy}$ powders.**

Brunauer-Emmett-Teller (BET) N_2 adsorption/desorption data

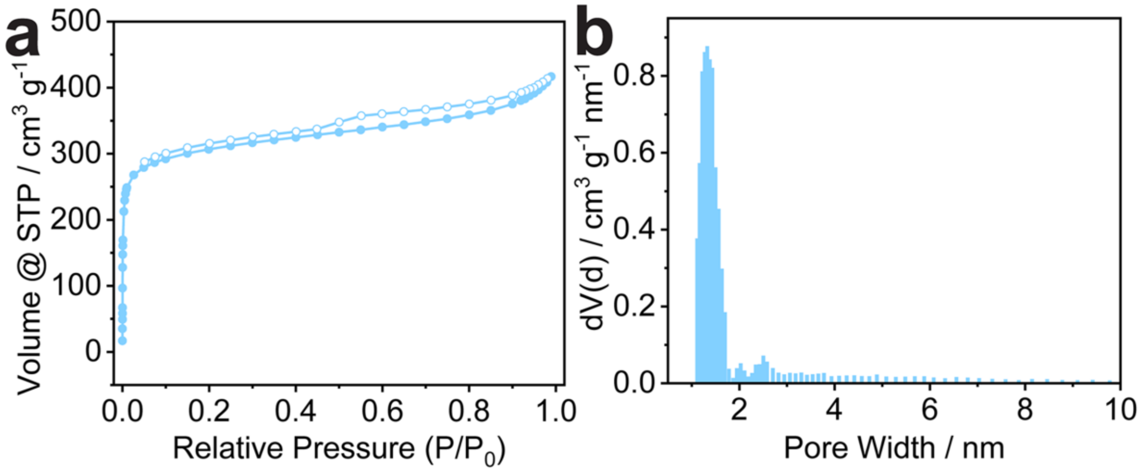

Supplementary Fig. 5. BET data of $COF_{TPDA\text{-}TFPy}$ powders. a N_2 adsorption/desorption isotherms of $COF_{TPDA\text{-}TFPy}$ powders at 77 K. **b** Pore size distribution (PSD) of $COF_{TPDA\text{-}TFPy}$ powders calculated using density functional theory (DFT).

Scanning Electron Microscope (SEM) images of $COF_{TPDA\text{-}TFPy}$ film

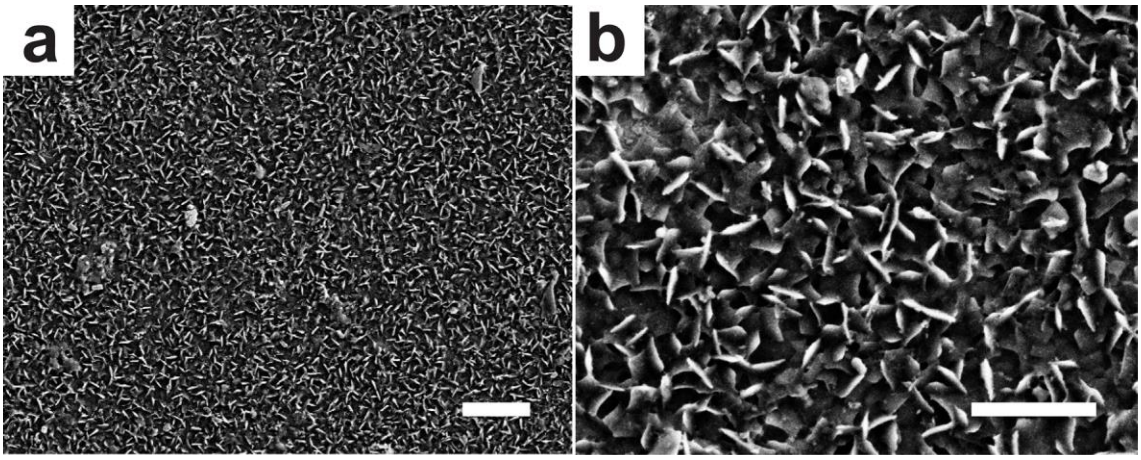

Supplementary Fig. 6. SEM images of $COF_{TPDA\text{-}TFPy}$. SEM images of $COF_{TPDA\text{-}TFPy}$ film in **a** low (Scale bar: 2 μm) and **b** high (Scale bar: 1 μm) magnification, respectively.

Atomic Force Microscope (AFM) images of $COF_{TPDA-TFPy}$ film

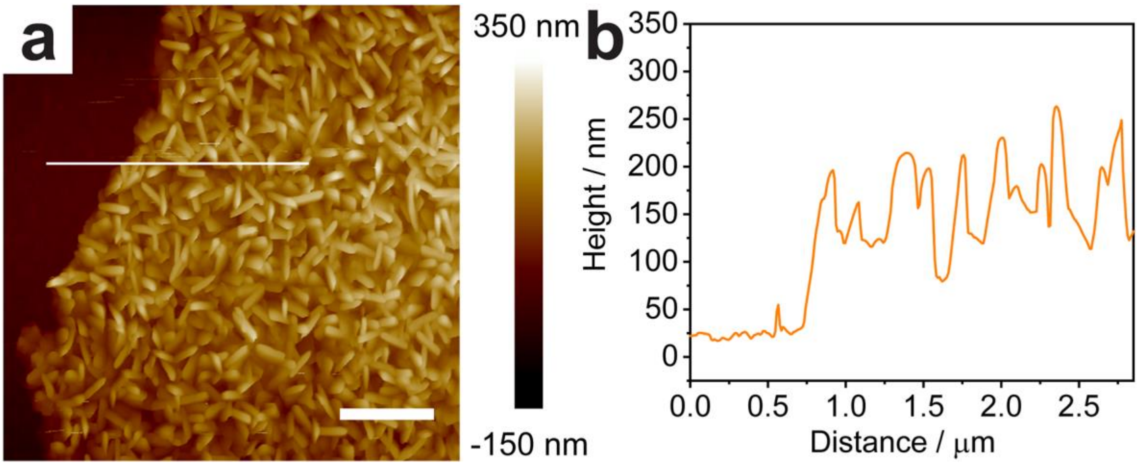

Supplementary Fig. 7. AFM images of $COF_{TPDA-TFPy}$. **a** AFM height image of $COF_{TPDA-TFPy}$ film on the glass substrate and **b** corresponding line profiles in a. (Scale bar: 1 μm)

Transmission Electron Microscope (TEM) images of $COF_{TPDA\text{-}TFPy}$ film

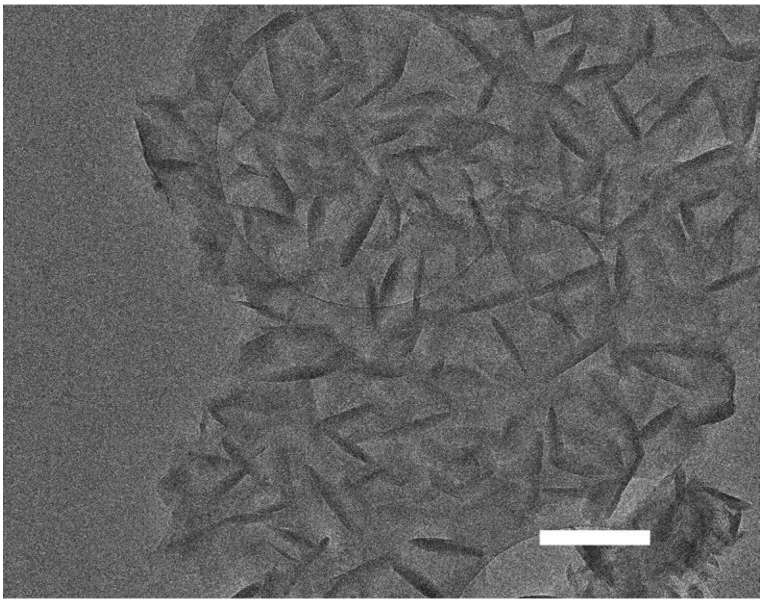

Supplementary Fig. 8. TEM images of $COF_{TPDA\text{-}TFPy}$. TEM images of $COF_{TPDA\text{-}TFPy}$ film in low magnification. (Scale bar: 500 nm)

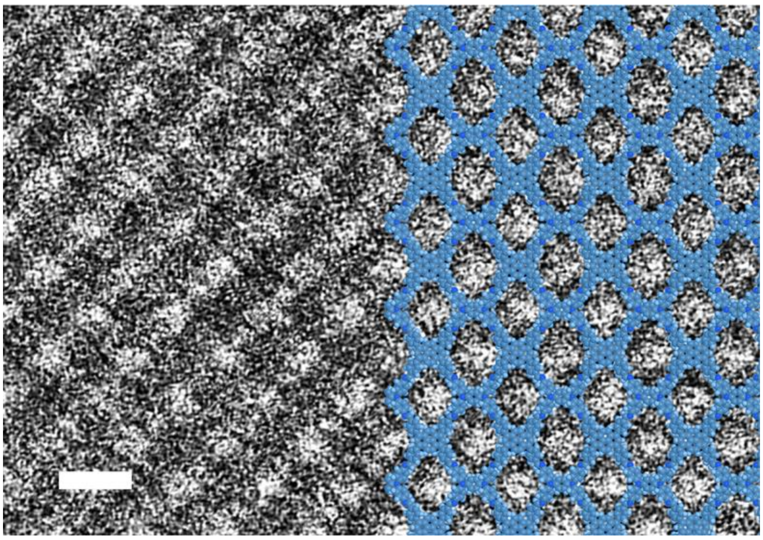

Supplementary Fig. 9. TEM images of $COF_{TPDA\text{-}TFPy}$. Left: Enlarged TEM image of $COF_{TPDA\text{-}TFPy}$ film. Right: Superimposed image of simulated $COF_{TPDA\text{-}TFPy}$ model on enlarged TEM image. (Scale bar: 2 nm)

Fingerprints and Sweat Pore Data

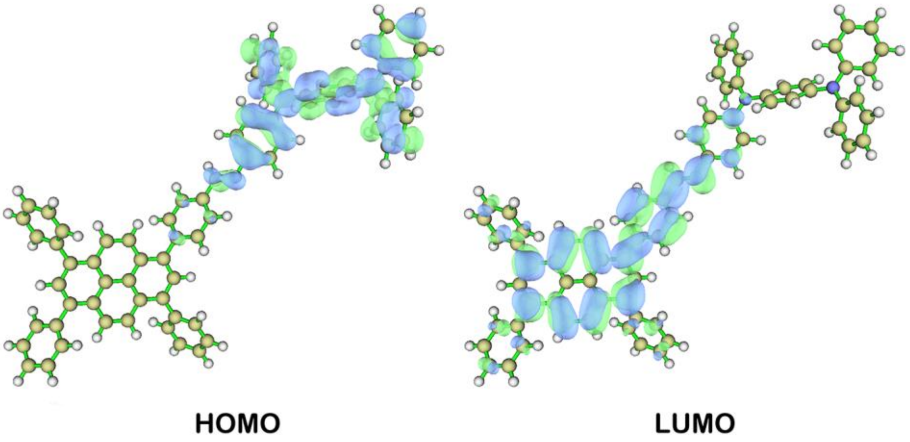

Supplementary Fig. 10. Calculated HOMO and LUMO charge density distributions of $COF_{TPDA\text{-}TFPy}$ segment. [1-2]

The frontier molecular orbital distribution of the $COF_{TPDA\text{-}TFPy}$ segment was calculated by DFT, b3lyp, 6–31G. The results of theoretical calculations revealed that the HOMO and LUMO of the $COF_{TPDA\text{-}TFPy}$ segment are generally located on the triphenylamine moiety and pyrene moiety respectively, which accords with the characteristics of a donor-acceptor structure.

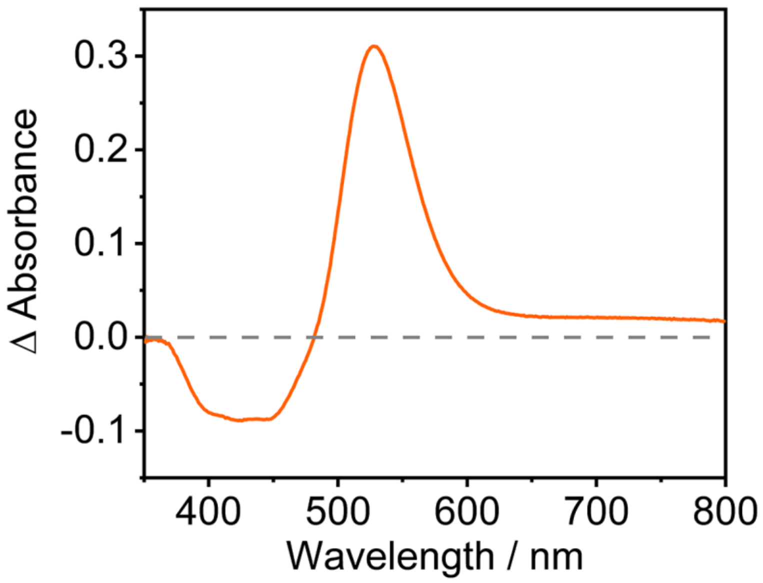

Supplementary Fig. 11. The wet/dry absorption differences. The absorption differences between $COF_{TPDA-TFPy}$ film at wet and dry states (Wet - Dry).

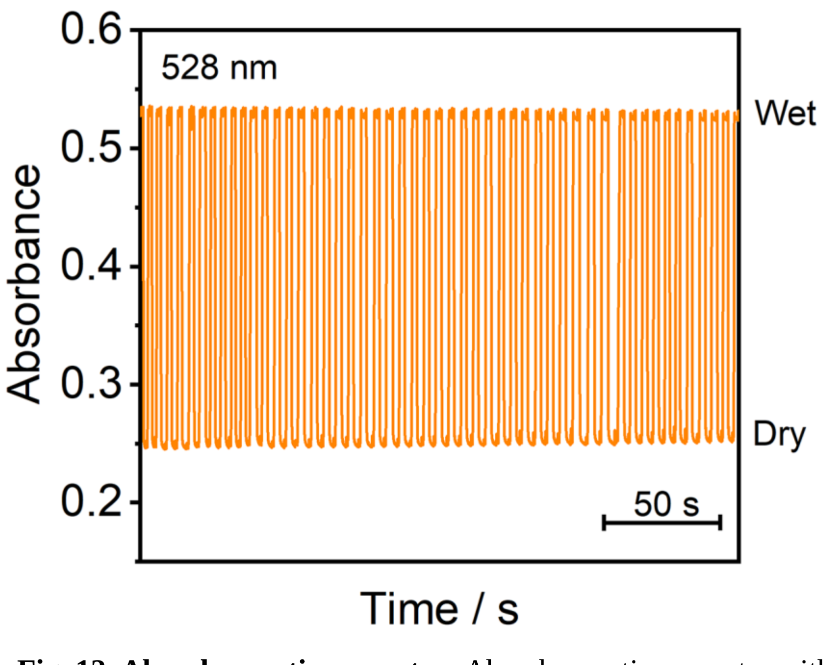

Supplementary Fig. 12. Absorbance-time spectra. Absorbance-time spectra within 50 cycles of hydrochromic switching by alternately exposing the $COF_{TPDA-TFPy}$ film to wet and dry N_2 stream. at 528 nm.

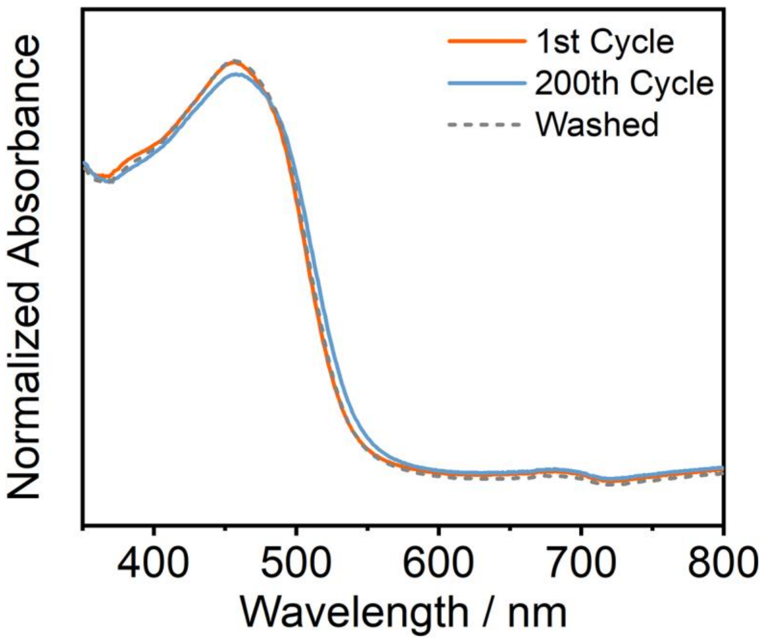

Supplementary Fig. 13. Absorbance spectra before and after washing. Absorption spectra of dry $COF_{TPDA-TFPy}$ film after the 1st (red) and 200th (blue) hydrochromic cycles. The dashed line is the absorption spectra of the $COF_{TPDA-TFPy}$ film after the 200th hydrochromic cycle and washed by ethanol.

Supplementary Table 1. The hydrochromic effect of solutions with different components with concentrations at sweat level.

Species	Components	Color after Drying
Salts	NaCl	Yellow
	KCl	Yellow
	$MgCl_2$	Yellow
	$CaCl_2$	Yellow
Small molecule	Glucose	Yellow
	Ascorbic Acid	Yellow
	Uric Acid	Yellow
	Lactic Acid	Grey
	Urea	Yellow
	Cysteine	Yellow
	Serine	Yellow
	Acetaminophen	Yellow
Acid/Base	NaOH	Yellow
	HCl	Grey
Protein	BSA	Red
	Dermcidin	Red

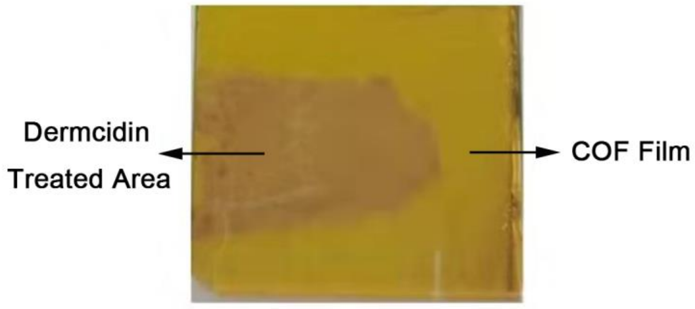

Supplementary Fig. 14. Solution-treated result. Photograph of the $COF_{TPDA-TFPy}$ film treated with 0.5 mg mL^{-1} dermcidin solution.

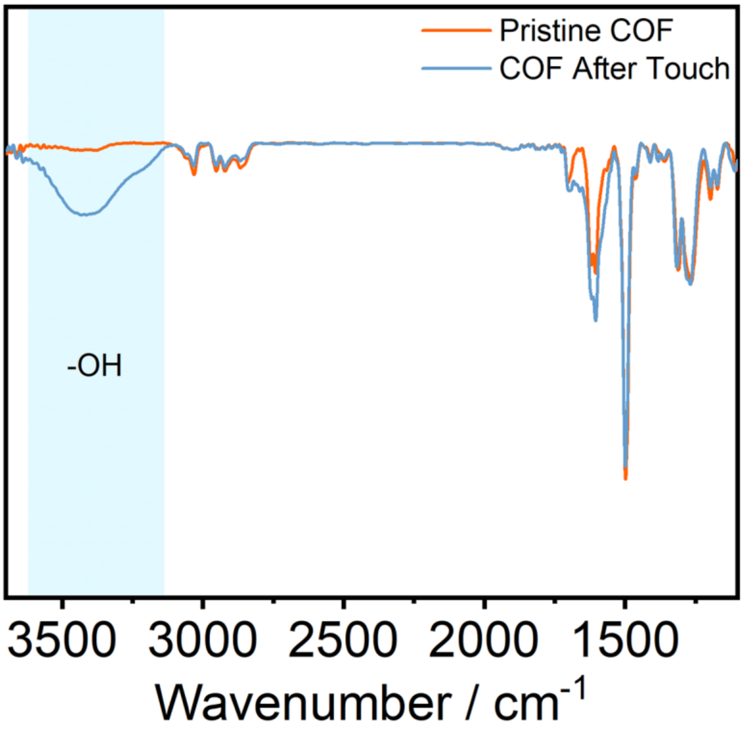

Supplementary Fig. 15. FTIR before and after touch. FTIR spectra of a $COF_{TPDA\text{-}TFPy}$ powder pellet before and after the touch of human fingers.

The FTIR spectra of a $COF_{TPDA\text{-}TFPy}$ powder pellet revealed that after the touch of human fingers, the remarkable peaks of -OH (3100~3600 cm^{-1}), which belonged to H_2O, emerged in the spectra of $COF_{TPDA\text{-}TFPy}$, which indicate the collection of the sweat of fingerprint residue.

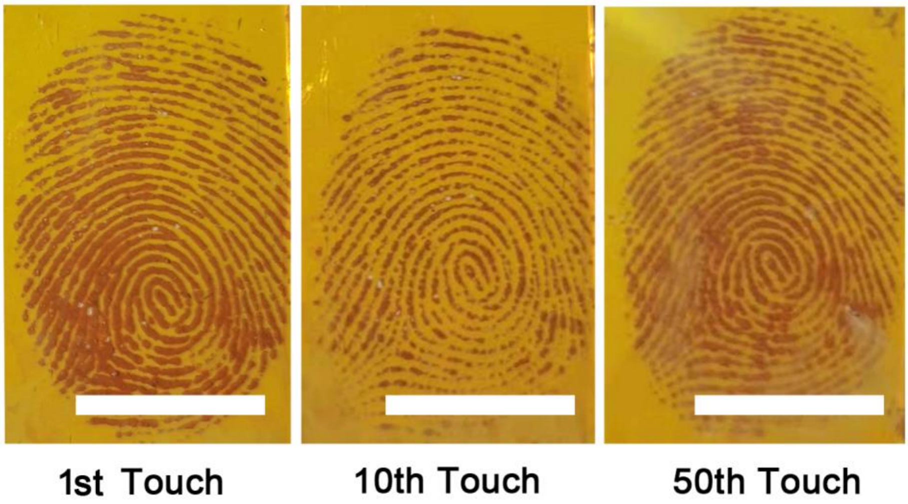

Supplementary Fig. 16. 50 times touch. Photographs of the sweat-induced fingerprints on the same $COF_{TPDA-TFPy}$ film at the 1st, 10th, and 50th collection operation. (Scale bars: 1cm)

The following calculations are based on the existing laboratory-level synthetic routes, and the costs would be further reduced through large-scale industrial production.

Monomers per batch: TPDA: 14.2 mg, 0.1 euros; TFPy: 18.6 mg, 2.7 dollars.

Solvents for per tube: o-dichlorobenzene: 3 mL, 0.34 dollars; n- n-butanol: 3mL, 0.33 dollars.

Glass substrates: 8 slices, 1.5 cm × 4.5 cm 0.64 dollars.

Considering electricity and other costs, the total cost is around 6 dollars for 1 tube.

Overall, for per collection (8 slices in 1 tube, and 1 slice can be reused 50 times), the cost is 6/8/50 = 0.015 dollars.

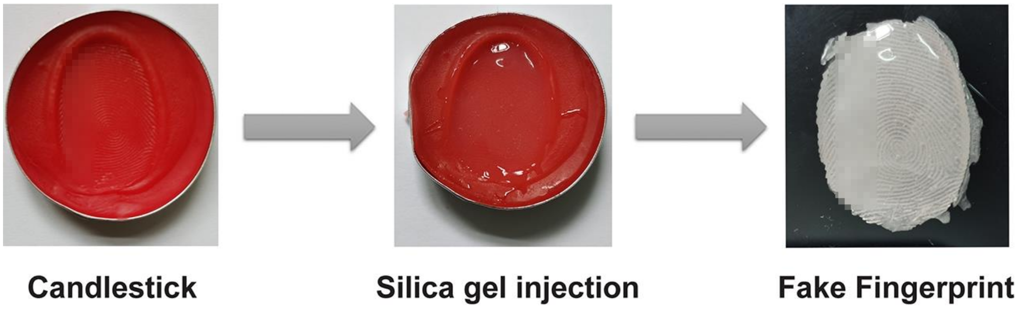

Supplementary Fig. 17. The preparation process of the fake fingerprint.

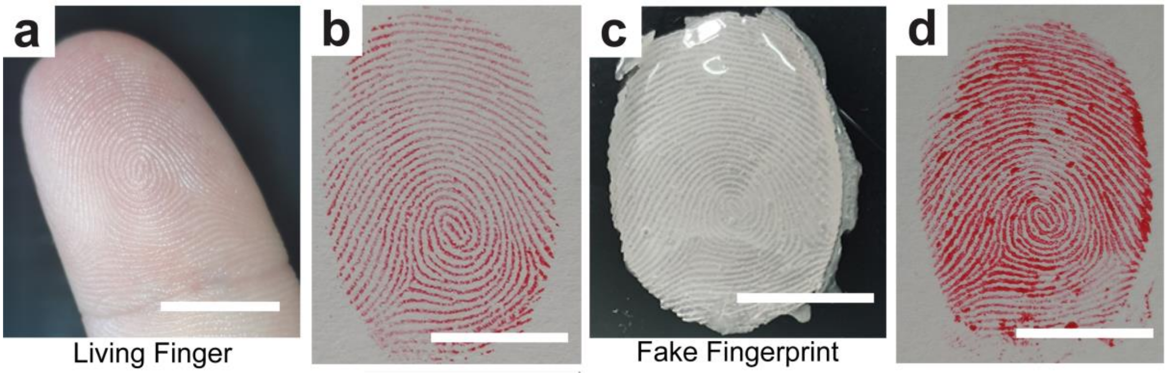

Supplementary Fig. 18. Fingerprints by coloured inks of a living finger and a fake fingerprint. Photographs of **a** a living finger and **c** a fake fingerprint from the finger in a. Fingerprint by colored inks of **b** the living finger and **d** fake fingerprint. (Scale bars: 1 cm)

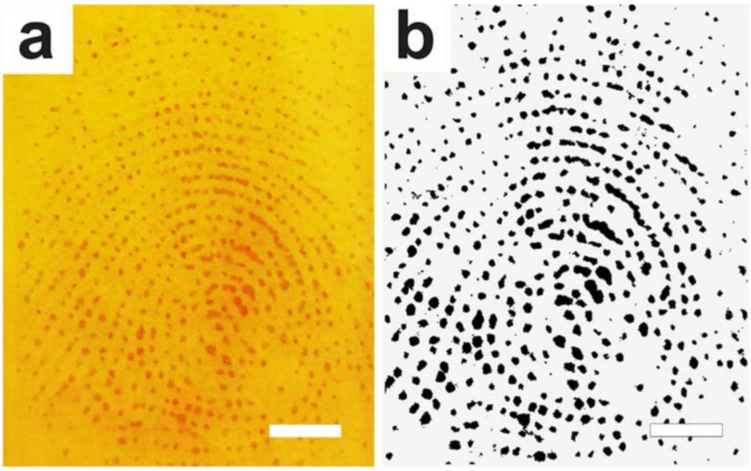

Supplementary Fig. 19. Sweat pore image from a finger. Picture of **a** another sweat pore distribution image and **b** extracted sweat pore distribution images (Added pseudocolors) from the same donor in Fig. 5. (Scale bars: 20 mm) The black (**b**) colored images are added pseudocolors by a Photoshop program for comparison purposes.

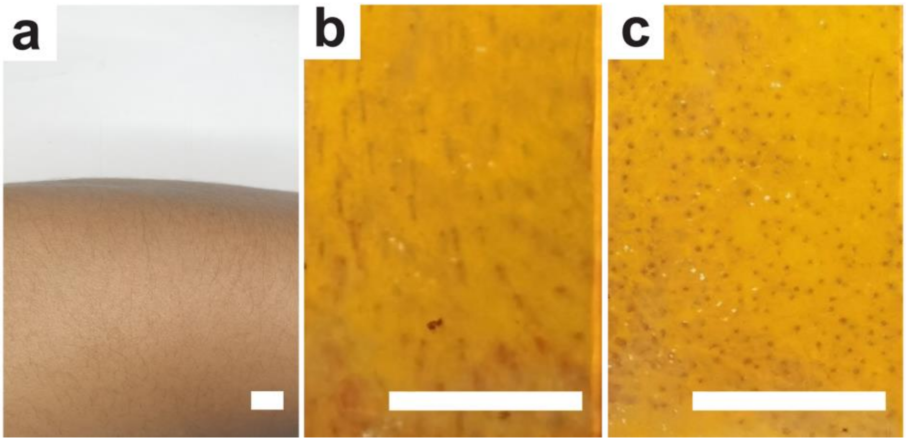

Supplementary Fig. 20. Sweat pore image from a hairy arm. Photographs of the **a** outside of an arm, and the sweat pore images collected from the outside of an arm before **b** and after shaving **c**, respectively. (Scale bars: 1 cm)

Supplementary Table 2. Similarity factors of another 60 fingerprint samples from FVC2002.

Sample	Similarity Factor	Sample	Similarity Factor	Sample	Similarity Factor	Sample	Similarity Factor
**102_5**	0.24056	**104_4**	0.25126	**106_3**	0.3118	**108_2**	0.26874
**102_6**	0.23426	**104_5**	0.20008	**106_4**	0.28868	**108_3**	0.25607
**102_7**	0.31277	**104_6**	0.3118	**106_5**	0.2466	**108_4**	0.22473
**102_8**	0.20851	**104_7**	0.21758	**106_6**	0.274	**108_5**	0.23868
**103_1**	0.22537	**104_8**	0.24311	**106_7**	0.28632	**108_6**	0.24533
**103_2**	0.23814	**105_1**	0.20333	**106_8**	0.27854	**108_7**	0.22398
**103_3**	0.20851	**105_2**	0.17791	**107_1**	0.26517	**108_8**	0.26275
**103_4**	0.24056	**105_3**	0.20333	**107_2**	0.3014	**109_1**	0.22048
**103_5**	0.26667	**105_4**	0.22048	**107_3**	0.25416	**109_2**	0.22453
**103_6**	0.24343	**105_5**	0.2321	**107_4**	0.2381	**109_3**	0.2357
**103_7**	0.20954	**105_6**	0.1701	**107_5**	0.25198	**109_4**	0.25198
**103_8**	0.24254	**105_7**	0.1972	**107_6**	0.28172	**109_5**	0.24759
**104_1**	0.30123	**105_8**	0.1857	**107_7**	0.19659	**109_6**	0.213
**104_2**	0.26261	**106_1**	0.35007	**107_8**	0.274	**109_7**	0.21664
**104_3**	0.28006	**106_2**	0.28006	**108_1**	0.20362	**109_8**	0.24343

Supplementary Table 3. Structural parameters of $COF_{TPDA-TFPy}$.

P2/m							
a = 23.5 Å, b = 22.8 Å, c = 4.3 Å, α =γ = 90°, β = 78°, Rwp = 7.6%, Rp = 6.1%							
**C1**	0.47102	1.60717	0.56188	**C18**	0.87586	0.09044	0.24604
**C2**	0.29292	1.71783	0.77461	**C19**	0.84858	0.05345	0.49172
**C3**	0.43924	1.55425	0.6388	**C20**	0.7954	0.07077	0.67706
**C4**	0.3788	1.55362	0.77246	**C21**	0.77077	0.12417	0.61925
**C5**	0.34547	1.60861	0.81595	**C22**	0.97009	0.05195	0.51491
**C6**	0.29467	1.61329	0.69041	**H23**	0.87594	0.17554	0.0222
**C7**	0.26816	1.66708	0.67349	**H24**	0.9159	0.07596	0.09213
**C8**	0.34087	1.71298	0.92128	**H25**	0.77408	0.04399	0.8762
**C9**	0.36574	1.65872	0.94993	**H26**	0.94894	0.09441	0.52571
**H10**	0.449	1.64917	0.6034	**C27**	0.72955	0.22467	0.27796
**H11**	0.27876	1.57463	0.58396	**H28**	0.72998	0.13855	0.76805
**H12**	0.23039	1.67178	0.56273	**H29**	0.70094	0.18676	0.25982
**H13**	0.35865	1.7523	1.01251	**C30**	0.35032	1.5	0.83329
**H14**	0.40236	1.6549	1.07009	**H31**	0.30403	1.5	0.9386
**N15**	0.78053	0.21818	0.33694	**C32**	0.46972	1.5	0.57114
**C16**	0.80068	0.1626	0.38983	**N33**	0.87654	0	0.54794
**C17**	0.85414	0.1455	0.20538	**C34**	0.93766	1	0.52456

Supplementary References

1. Xiao, H., Wu, H. & Chi, X. SCE: grid environment for scientific computing in *Networks for Grid Applications,* Vol. 2 (Eds: P. V.-B. Primet, T. Kudoh, J. Mambretti), 35-42 (2009)
2. Lu, T. & Chen, F. Multiwfn: a multifunctional wavefunction analyzer. *J. Comput. Chem.* **33**, 580-592 (2012).
