# Supplementary material for: A sweat-responsive covalent organic framework film for material-based liveness detection and sweat pore analysis
Source: Nat Commun. 2023 Feb 3;14:578. doi: 10.1038/s41467-023-36291-9 (PMC9894872; doi:10.1038/s41467-023-36291-9)
Supplement: Supplementary file 2 — Description of Additional Supplementary Files [file 41467_2023_36291_MOESM2_ESM.pdf]

Description of Additional Supplementary Files

File Name: Supplementary Data 1:

Description: The 12 fingerprint samples of 1 donor in this manuscript.

File Name: Supplementary Movie 1:

Description: Collection of sweat fingerprints.

File Name: Supplementary Movie 2:

Description: Breathing hydrochromism of COF film.

File Name: Supplementary Movie 3:

Description: Collection of sweat pore images.
