# Supplementary figures and images for: A sweat-responsive covalent organic framework film for material-based liveness detection and sweat pore analysis
Source: Nat Commun. 2023 Feb 3;14:578. doi: 10.1038/s41467-023-36291-9 (PMC9894872; doi:10.1038/s41467-023-36291-9)

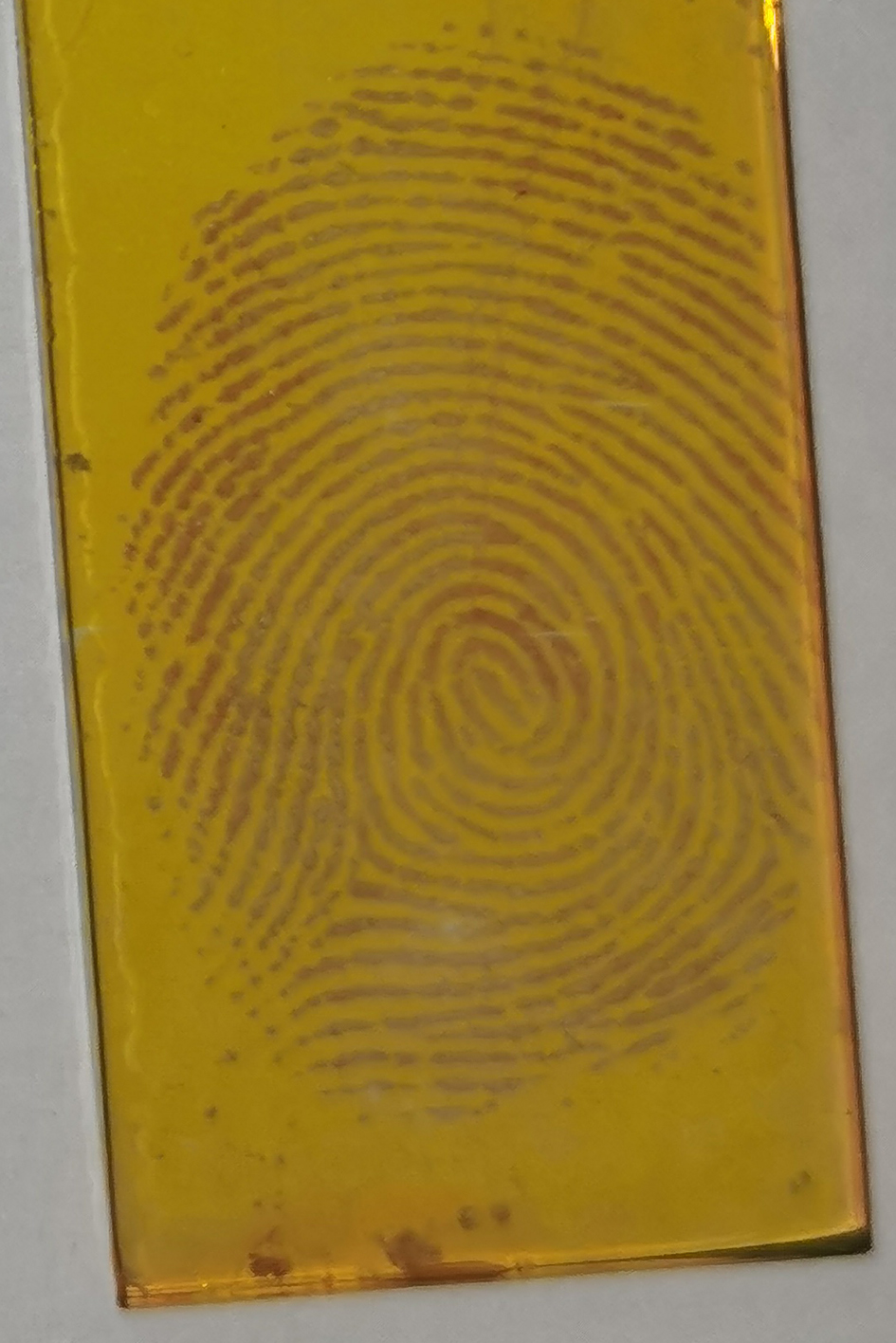

Supplement: Supplementary file 3 — Supplementary Data 1 [file 41467_2023_36291_MOESM3_ESM.zip › 101.jpg]

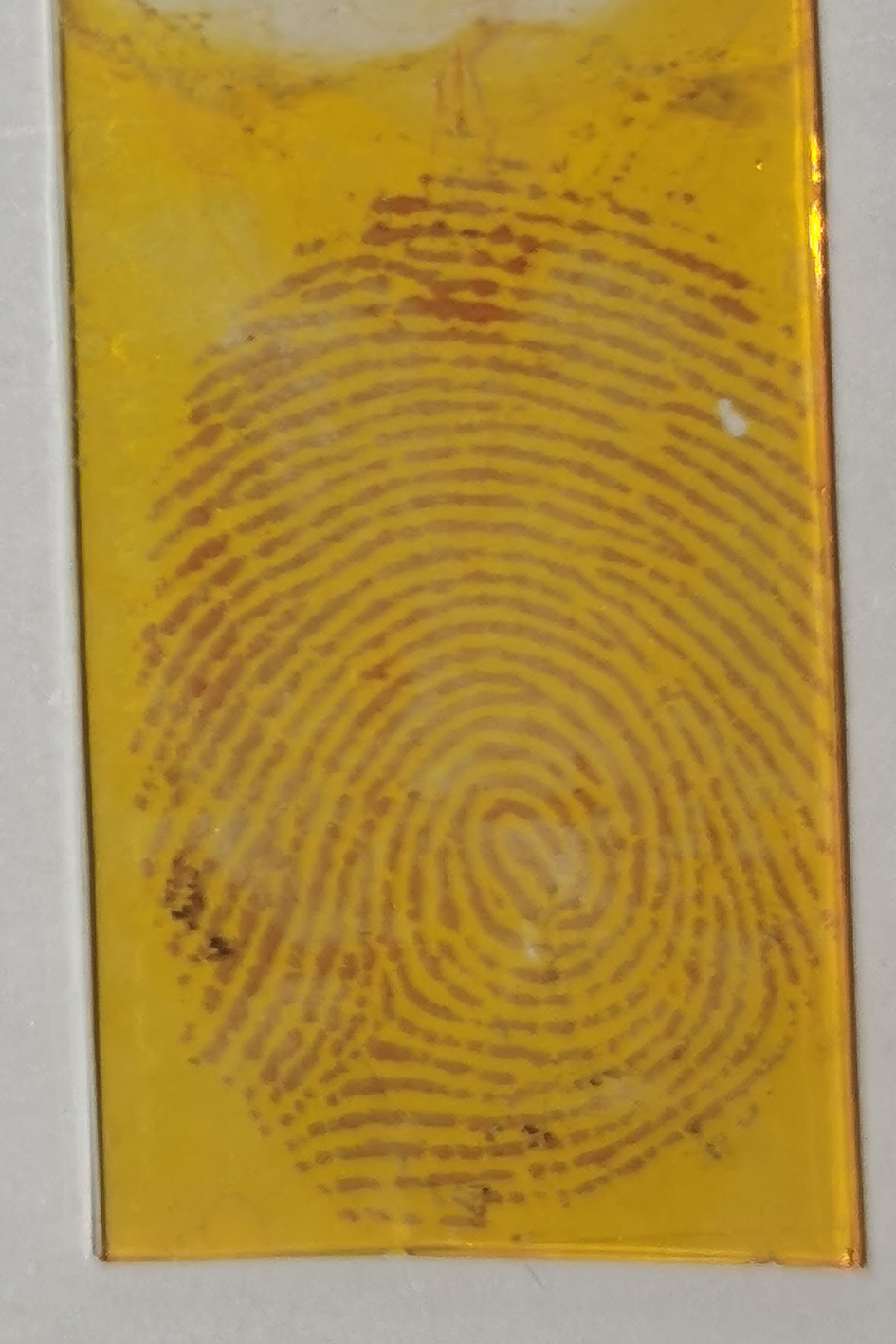

Supplement: Supplementary file 3 — Supplementary Data 1 [file 41467_2023_36291_MOESM3_ESM.zip › 102.jpg]

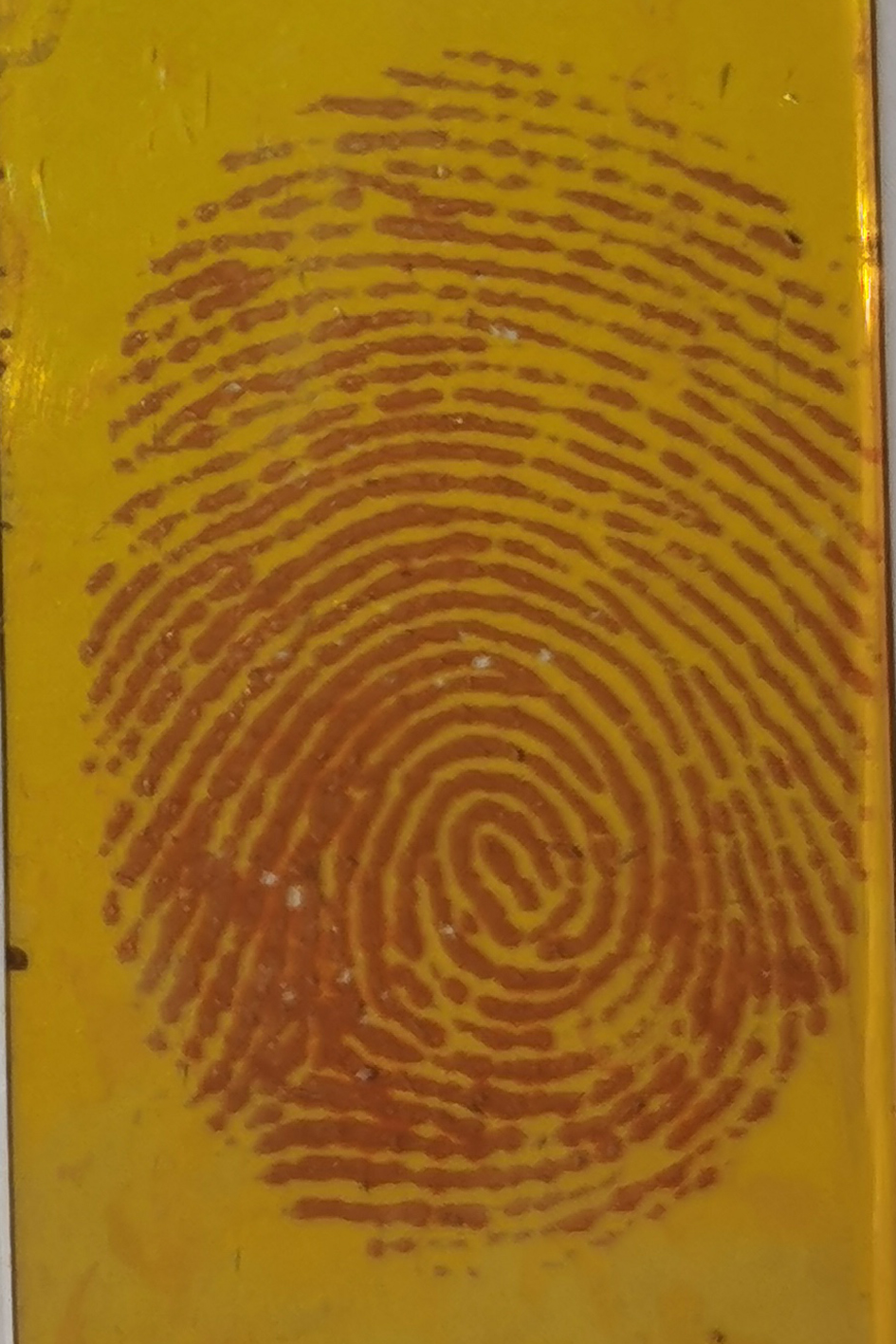

Supplement: Supplementary file 3 — Supplementary Data 1 [file 41467_2023_36291_MOESM3_ESM.zip › 102_1.jpg]

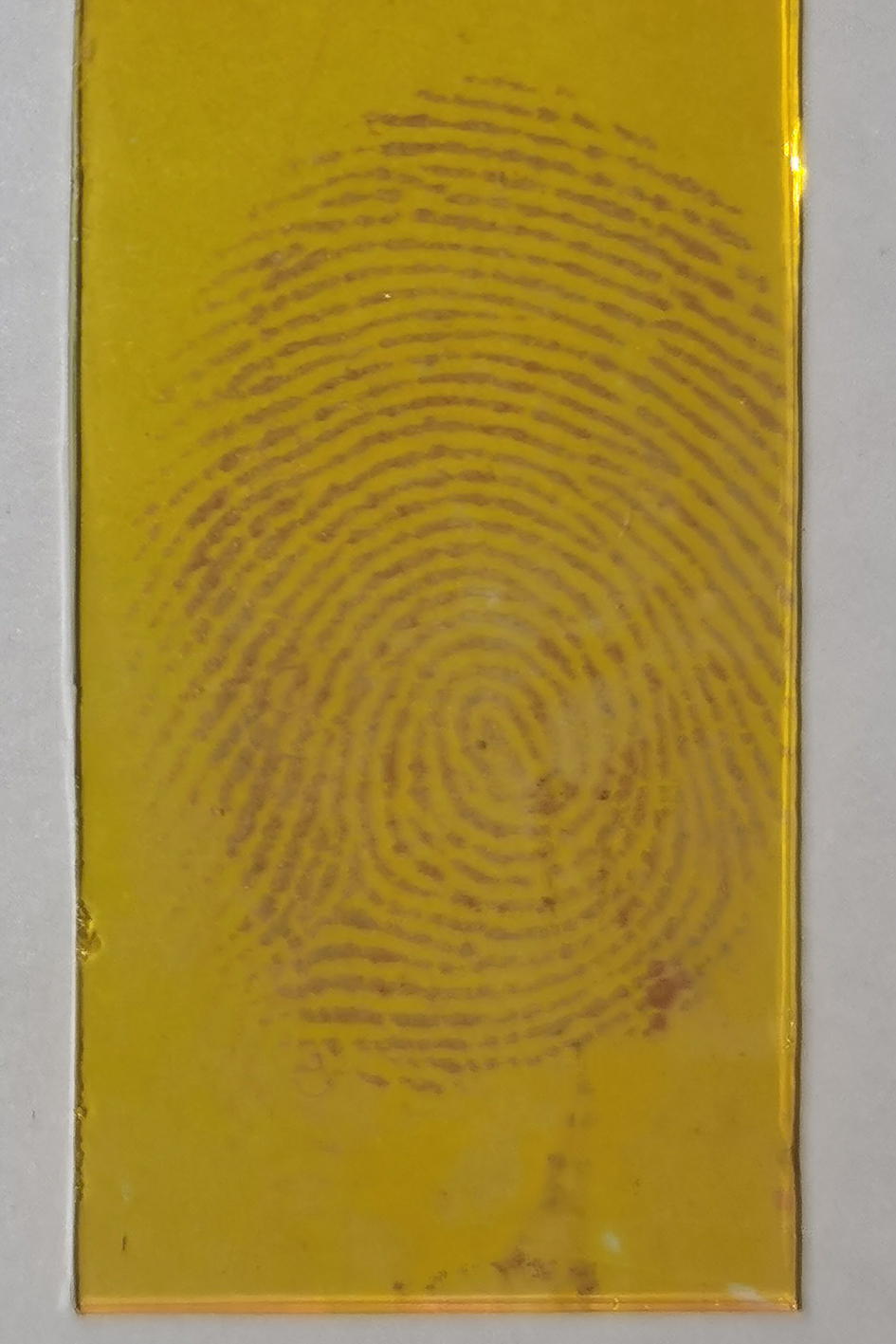

Supplement: Supplementary file 3 — Supplementary Data 1 [file 41467_2023_36291_MOESM3_ESM.zip › 102_2.jpg]

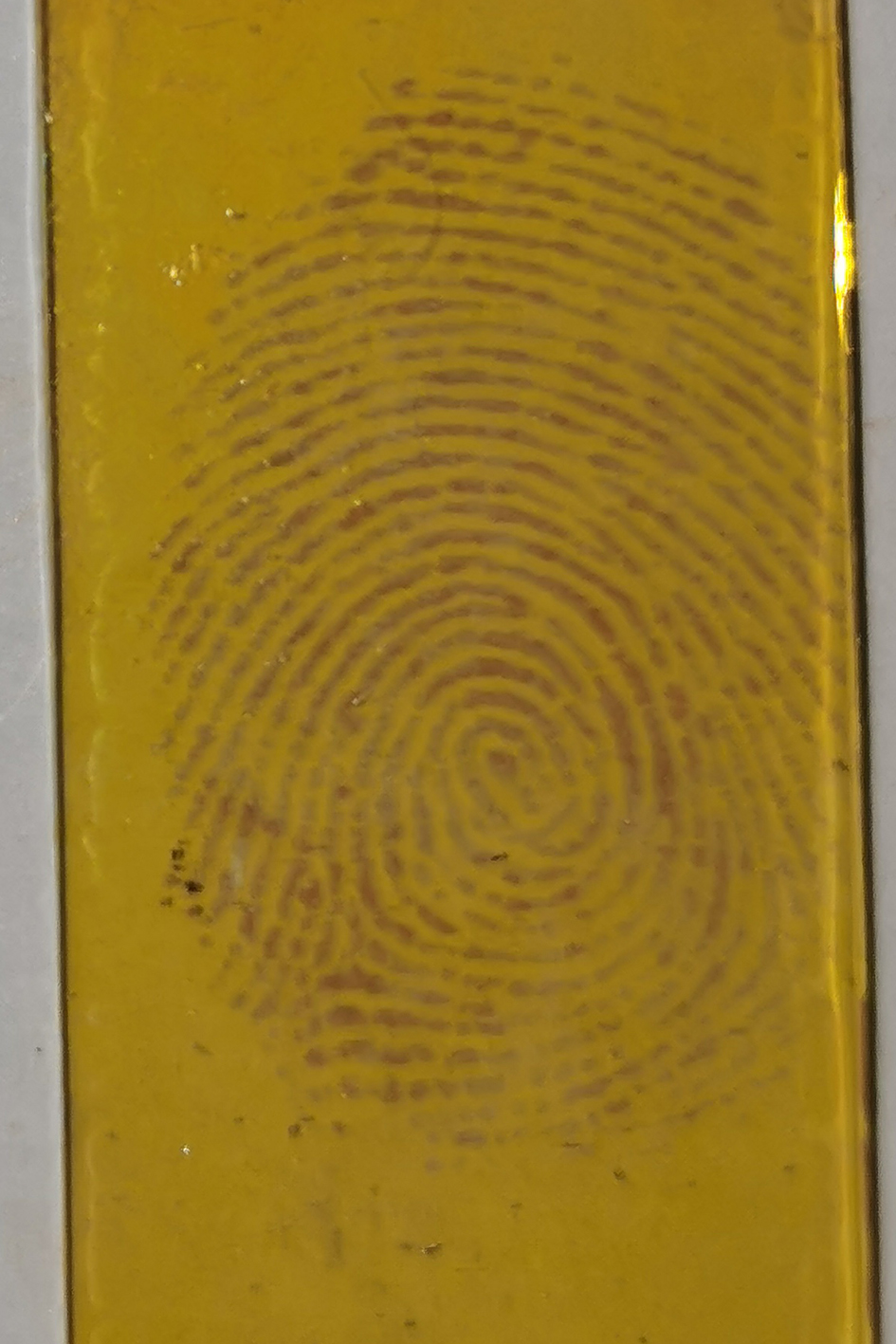

Supplement: Supplementary file 3 — Supplementary Data 1 [file 41467_2023_36291_MOESM3_ESM.zip › 102_3.jpg]

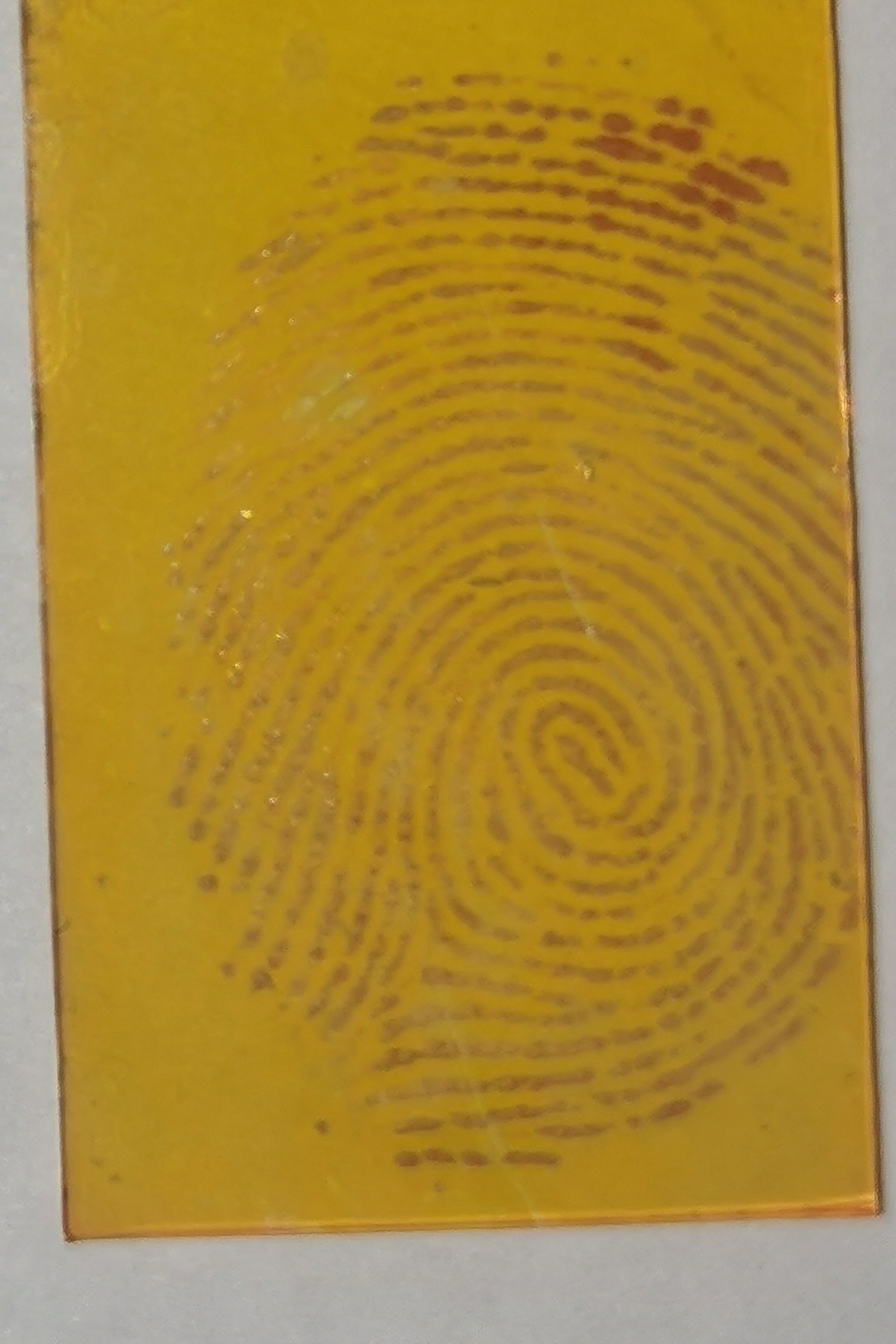

Supplement: Supplementary file 3 — Supplementary Data 1 [file 41467_2023_36291_MOESM3_ESM.zip › 102_4.jpg]

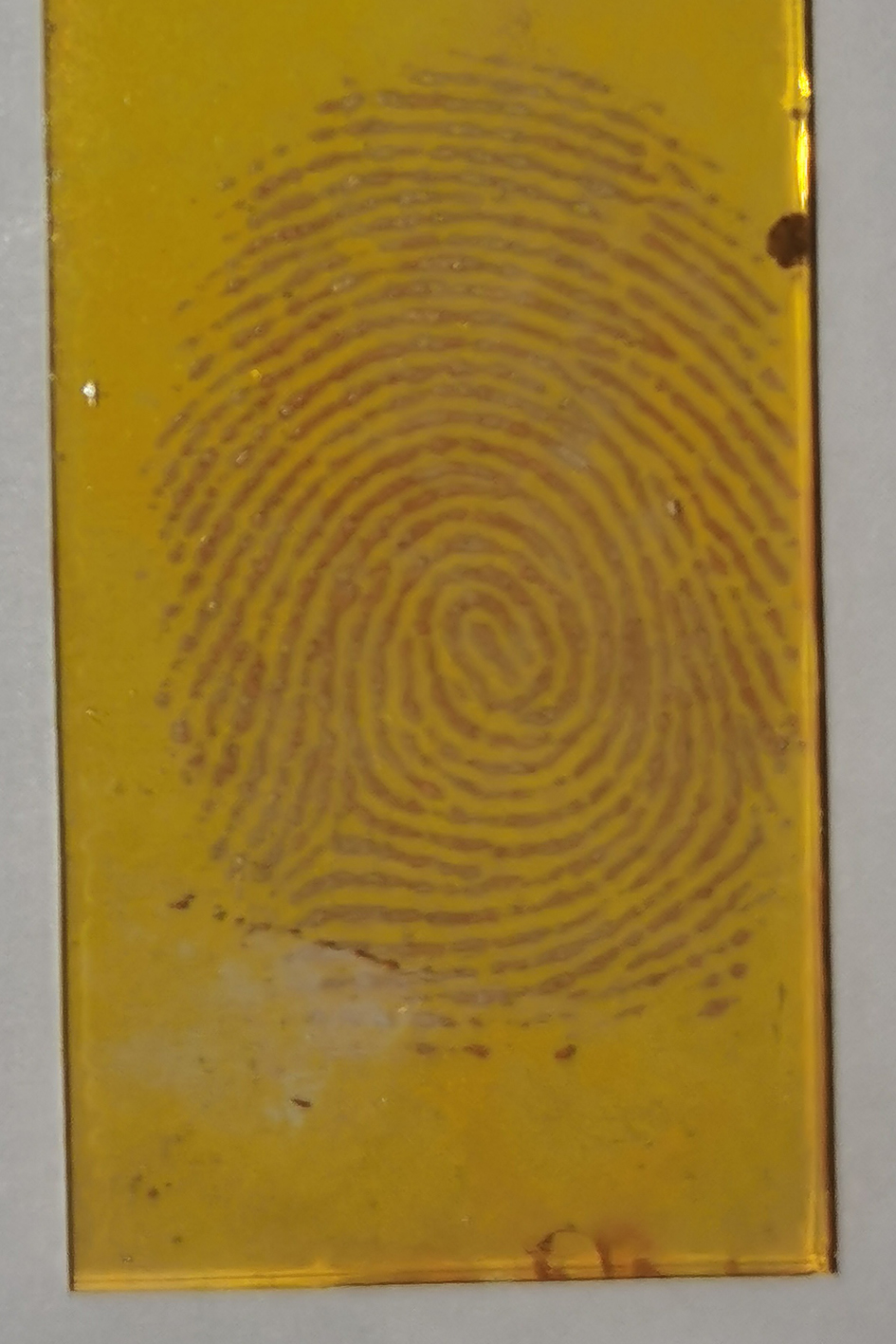

Supplement: Supplementary file 3 — Supplementary Data 1 [file 41467_2023_36291_MOESM3_ESM.zip › 103.jpg]

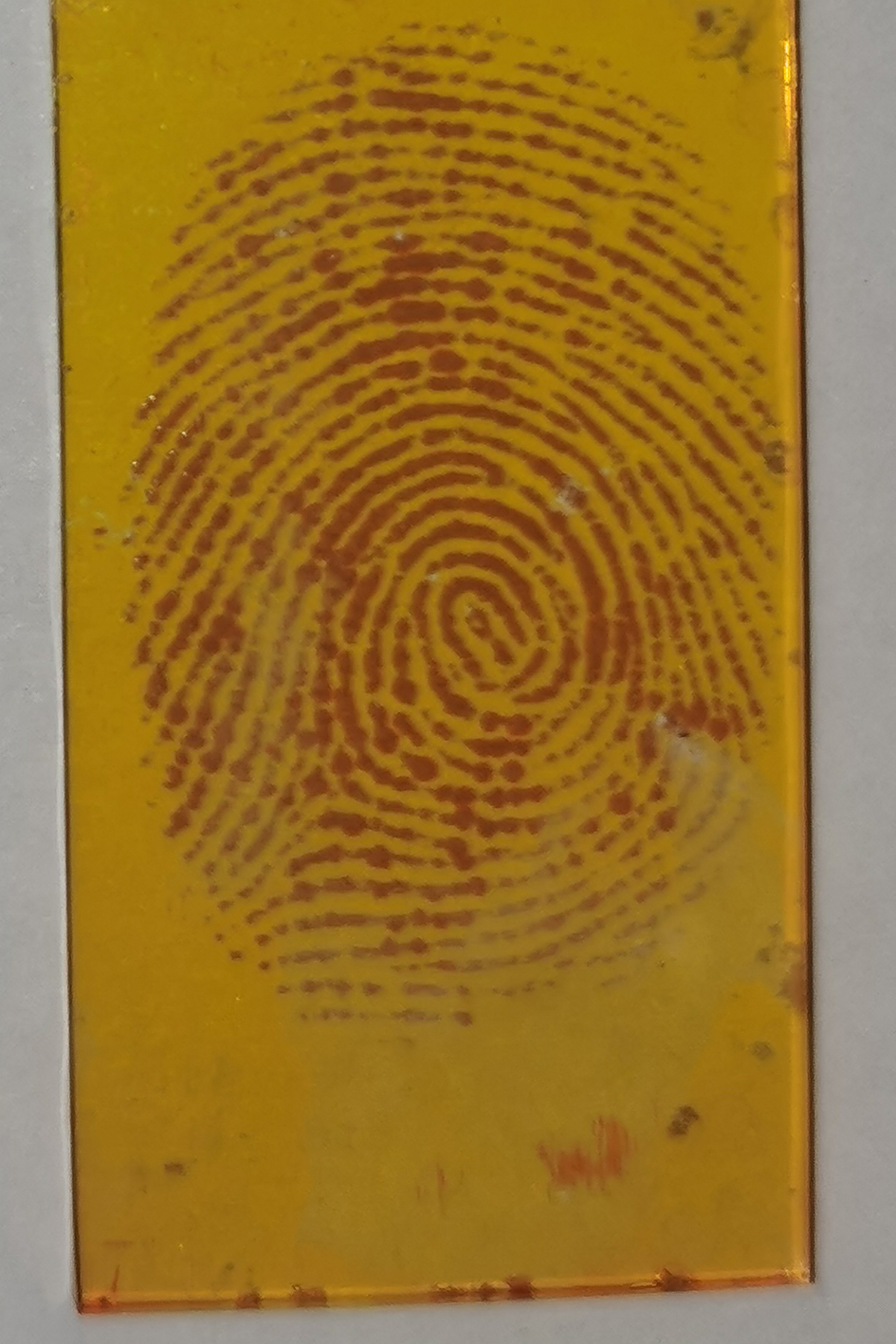

Supplement: Supplementary file 3 — Supplementary Data 1 [file 41467_2023_36291_MOESM3_ESM.zip › 104.jpg]

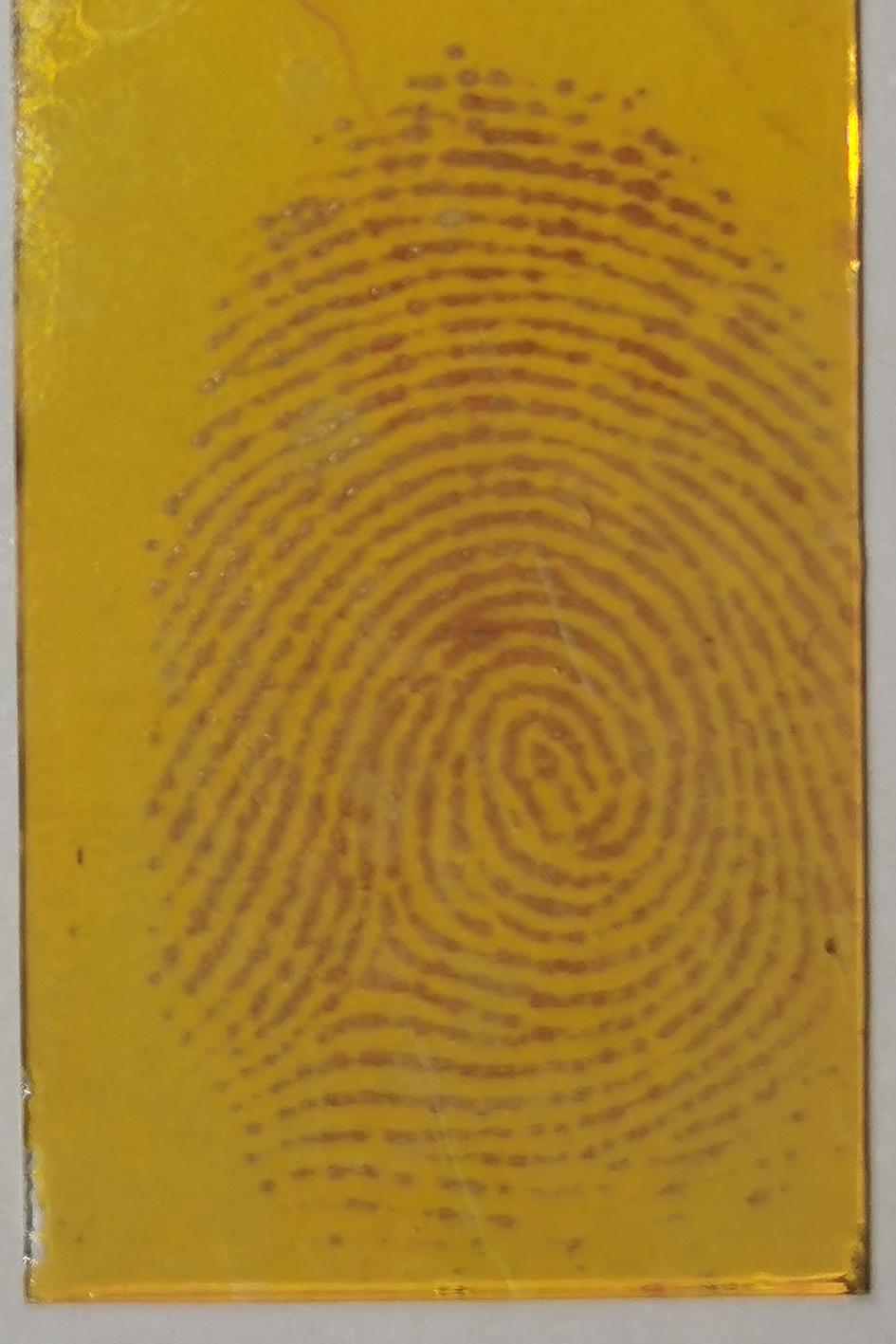

Supplement: Supplementary file 3 — Supplementary Data 1 [file 41467_2023_36291_MOESM3_ESM.zip › 105.jpg]

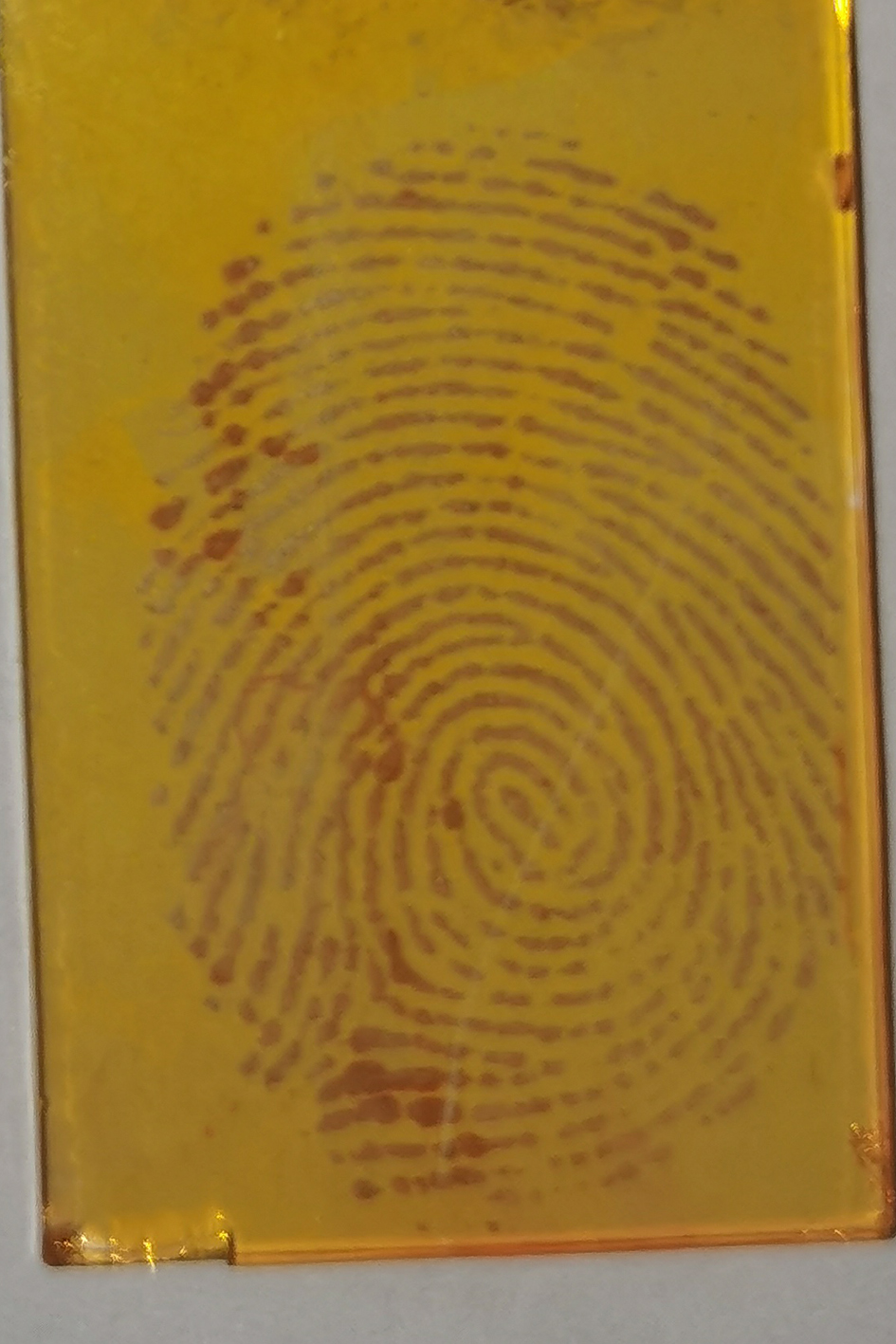

Supplement: Supplementary file 3 — Supplementary Data 1 [file 41467_2023_36291_MOESM3_ESM.zip › 106.jpg]

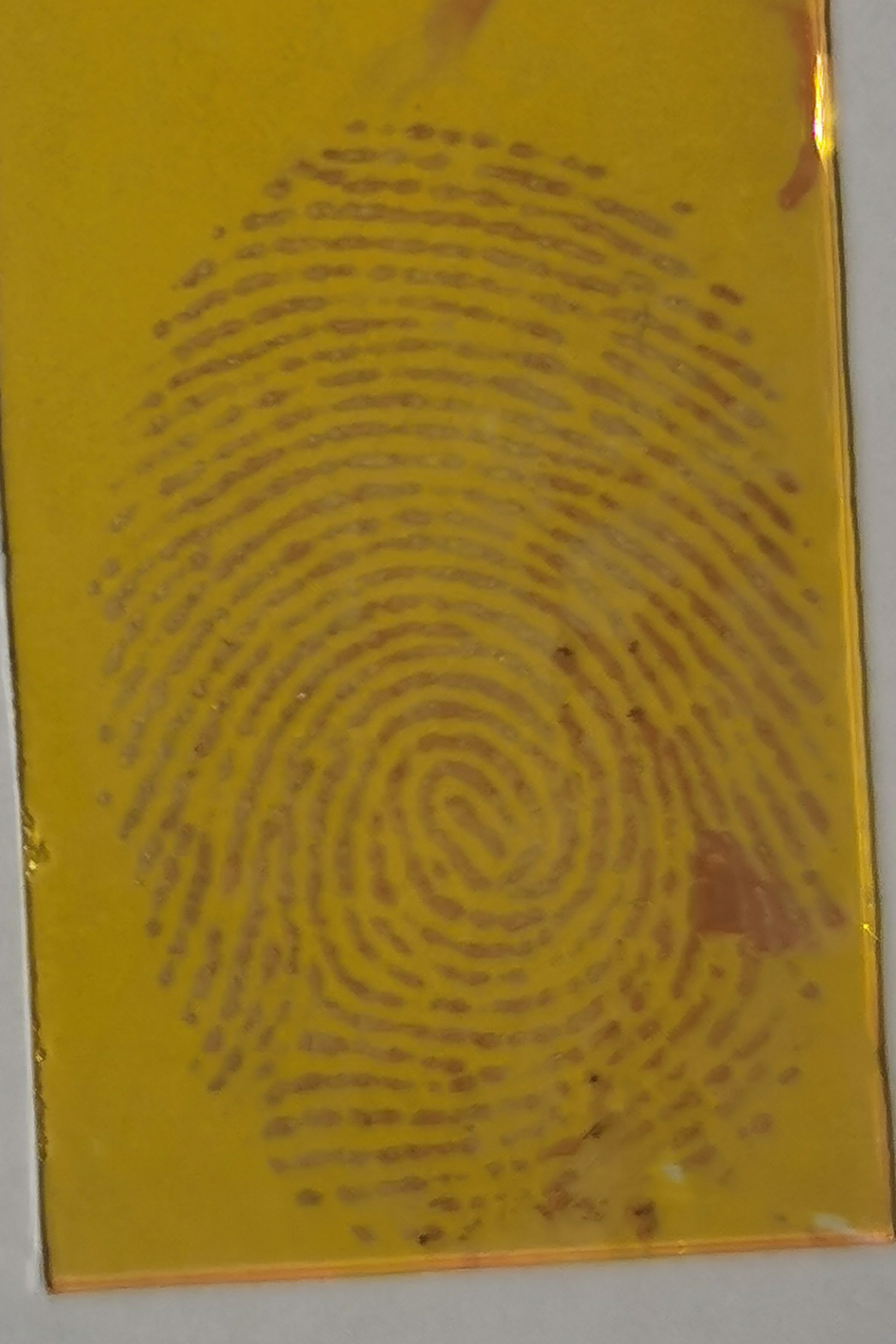

Supplement: Supplementary file 3 — Supplementary Data 1 [file 41467_2023_36291_MOESM3_ESM.zip › 107.jpg]

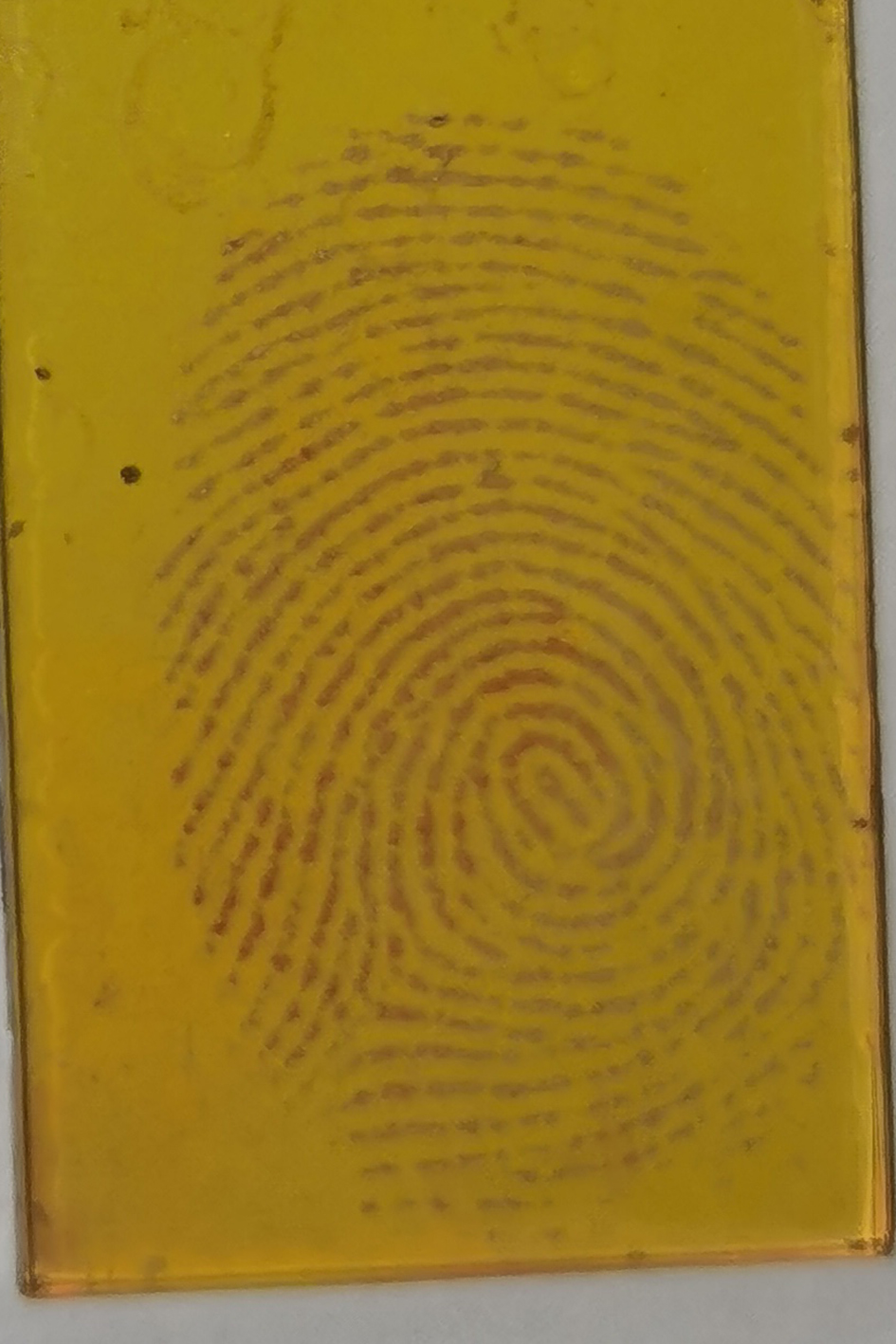

Supplement: Supplementary file 3 — Supplementary Data 1 [file 41467_2023_36291_MOESM3_ESM.zip › 108.jpg]
